# Broad-Spectrum Gramicidin S Derivatives with Potent Activity Against Multidrug-Resistant Gram-Negative ESKAPE Pathogens

**DOI:** 10.3390/antibiotics14050423

**Published:** 2025-04-22

**Authors:** John T. Kalyvas, Yifei Wang, Ornella Romeo, John R. Horsley, Andrew D. Abell

**Affiliations:** 1 Department of Chemistry, School of Physics, Chemistry and Earth Sciences, The University of Adelaide, Adelaide, SA 5005, Australia; john.kalyvas@adelaide.edu.au (J.T.K.); yifei.wang@adelaide.edu.au (Y.W.); john.horsley@adelaide.edu.au (J.R.H.); 2 Research Centre for Infectious Diseases, School of Biological Sciences, The University of Adelaide, Adelaide, SA 5005, Australia; ornella.romeo@adelaide.edu.au

**Keywords:** antibiotic resistance, antimicrobial peptides, antibiotics, gramicidin S, broad-spectrum, ESKAPE pathogens

## Abstract

Background/Objectives: Multidrug-resistant Gram-negative ESKAPE pathogens, including *E. coli*, *K. pneumoniae*, *P. aeruginosa*, and *A. baumannii*, pose a significant global health threat. Gramicidin S, a potent cyclic antimicrobial peptide, is largely ineffective against these bacteria, and its high haemolytic toxicity limits its clinical usage. This study reports on several novel gramicidin S analogues with improved efficacy and safety profiles against multidrug-resistant Gram-negative bacteria. Methods: A total of 19 gramicidin S derivatives were synthesised using Fmoc-based solid-phase peptide synthesis with targeted substitutions to enhance cationicity and modulate hydrophobicity. Minimum inhibitory concentrations (MICs) were determined against standard Gram-negative and Gram-positive strains. Haemolytic toxicity and in vitro nephrotoxicity were evaluated using human red blood cells and HEK-293 cells, respectively. All peptides were characterised by RP-HPLC and HRMS. Results: The selective incorporation of ^D^Arg and Trp significantly enhanced activity against Gram-negative bacteria while reducing cytotoxicity. Peptide **8** improved the therapeutic index (TI) against *E. coli* by 10-fold (MIC: 8 µg/mL; TI: 4.10) compared to gramicidin S (MIC: 32 µg/mL; TI: 0.38). Peptide **9** exhibited an 8-fold potency increase against *K. pneumoniae* and a 25-fold TI improvement. Peptide **19** enhanced activity against *P. aeruginosa* 8-fold over gramicidin S, while peptide **7** showed a 27-fold TI enhancement. All active peptides retained broad-spectrum activity against *S. aureus*, including MRSA. Conclusions: The findings highlight the critical role of balancing hydrophobicity and cationicity to overcome species-specific resistance mechanisms. Our gramicidin S analogues demonstrate potent broad-spectrum activity with significantly reduced toxicity compared to the parent peptide, providing a robust platform for the development of new antibiotics against ESKAPE bacterial pathogens.

## 1. Introduction

Gram-negative bacteria present a formidable challenge in clinical settings due to their inherently resilient structural features and high levels of antibiotic resistance [1]. These species possess an additional outer membrane rich in lipopolysaccharides that acts as a formidable barrier to many antibiotics [2]. The membrane not only impedes penetration but also houses efflux pumps and enzymes capable of degrading antibiotics [3,4]. Among the ESKAPE pathogens—an acronym for *Enterococcus faecium*, *Staphylococcus aureus*, *Klebsiella pneumoniae*, *Acinetobacter baumannii*, *Pseudomonas aeruginosa*, and *Enterobacter* species—Gram-negative strains are responsible for a significant proportion of global community and hospital-acquired infections, leading to increased morbidity and mortality rates [5].

Critically, these Gram-negative pathogens are now developing resistance to frontline antibiotics, such as carbapenems [6], underscoring the need for new antibacterial agents. In this context, antimicrobial peptides are a promising alternative to combat drug resistance. These naturally occurring molecules are found in a wide range of organisms, from microorganisms to humans, and are an integral component of the innate immune system [6]. The mechanism of action for antimicrobial peptides fundamentally differs from that of conventional antibiotics, as they primarily target the bacterial membrane, leading to disruption and subsequent cell death [7]. This membrane-targeting ability makes it challenging for bacteria to develop resistance [8], as it would require significant alterations to membrane composition, a costly adaptation in terms of energy and resources.

While we [9,10] and others [11] have previously shown that high hydrophobicity within antimicrobial peptides is crucial for eliminating Gram-positive bacteria, high cationicity is critical to effectively target Gram-negative pathogens [12]. The FDA-approved antibiotics polymyxin B and colistin are cationic peptides that are most commonly used as a last line of defence against infections caused by these pathogenic bacteria [13]. However, their significant nephrotoxicity and associated adverse effects limit their clinical application [6], and several bacterial strains are now becoming resistant to these last-resort antibiotics [8,13]. This has led to renewed interest in gramicidin S [14,15], a naturally occurring cyclic FDA-approved antimicrobial peptide known to target several cellular components, including the cell membrane [16]. Although gramicidin S has long been restricted to topical use due to its high haemolytic cytotoxicity, no cases of acquired resistance have been reported in the literature to date [17]. Gramicidin S primarily targets Gram-positive bacteria [18], including methicillin-resistant *S. aureus* (MRSA), but its limited spectrum of activity restricts its broader therapeutic applications. We postulate that enhancing the cationic charge of gramicidin S and fine-tuning global hydrophobicity would strengthen interactions with the negatively charged bacterial outer membrane of Gram-negative bacteria [19] to facilitate membrane disruption, thereby broadening the antibacterial spectrum for greater efficacy against these resilient pathogens.

Although *A. baumannii*, *E. coli*, *P. aeruginosa*, and *K. pneumoniae* are phenotypically similar in terms of their antimicrobial resistance profiles and metabolism, they represent a heterogeneous group of pathogens with significant variation in virulence mechanisms, primary sites of infection, and outer membrane architecture [1]. Hence, these structural and functional differences underscore the importance of targeted approaches in the rational design of peptide-based antibiotics.

## 2. Results and Discussion

The antimicrobial activity of several novel gramicidin S derivatives [9,10] was evaluated against a panel of benchmark Gram-negative clinical isolates, *A. baumannii* ATCC 19606, *E. coli* ATCC 25922, *P. aeruginosa* ATCC 27853, and *K. pneumoniae* ATCC 33455 in vitro, with haemolytic toxicity determined against human red blood cells.

*Acinetobacter baumannii* (*A. baumannii*) is a critical-priority pathogen due to its extreme resistance to frontline carbapenem antibiotics [20] and the persistence of highly virulent strains in hospital environments [21,22]. The notable resistance profile of the bacteria is further compounded by the capacity to form robust biofilms, which act as physical barriers to protect bacterial cells from antibiotics and the immune system [22]. These biofilms, composed of polysaccharides, proteins, lipids, and extracellular DNA, form a hydrophobic matrix that facilitates strong adhesion to hydrophobic surfaces [22]. Hence, it is likely that hydrophobic antibacterial peptides that exploit a similar affinity-based mechanism may enable deeper biofilm penetration and enhance antibacterial efficacy.

**Figure 1:**
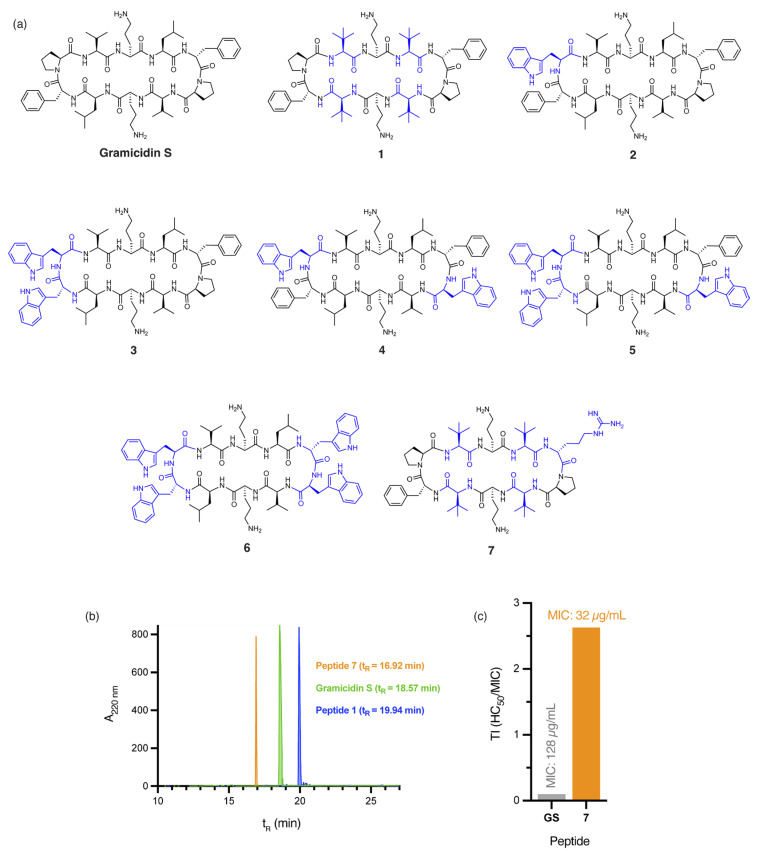
(**a**) Structure of gramicidin S and analogues **1**–**7**, with residues highlighted in blue indicating substitutions relative to the parent compound. (**b**) RP-HPLC traces for peptides **1**, **7**, and gramicidin S. (**c**) Calculated therapeutic indices (TI) for gramicidin S (GS) and peptide **7** against *E. coli*. Therapeutic index (TI) is defined as the ratio of the toxic dose (HC_50_) to the effective dose (MIC) of a drug, indicating its safety margin.

The incorporation of four non-proteinogenic Tle residues, as in peptide **1** (Figure 1a), significantly increases global hydrophobicity relative to gramicidin S, as demonstrated by predicted logP values (peptide **1**: logP = 2.01, gramicidin S: logP = 1.23) calculated by the Crippen method [23] and RP-HPLC retention times (peptide **1**: t_R_ = 19.90 min, gramicidin S: t_R_ = 18.57 min, Figure 1b). Not surprisingly, a two-fold increase in antibacterial activity against *A. baumannii* was observed for peptide **1** (MIC: 4 µg/mL, Table 1) compared to gramicidin S (MIC: 8 µg/mL, Table 1). However, the high haemolytic toxicity of **1** (HC_50_: 5.90 ± 0.23 µg/mL) affords an extremely poor therapeutic index (TI = 1.48). Incorporating ^D^Trp/Trp residues into the β-turn region(s) of gramicidin S further increases hydrophobicity, as evidenced by the calculated logP values of peptides **2**–**6** (Figure 1a)—peptide **2** (logP = 2.45), **3** (logP = 2.93), **4** (logP = 3.66), **5** (logP = 4.14), and **6** (logP = 4.63)—compared to gramicidin S (logP = 1.23). Specifically, as the calculated logP values of the peptides increased, antibacterial activity against *A. baumannii* progressively decreased—peptide **2** (MIC: 64 µg/mL), **3** (MIC: 128 µg/mL), **4** (MIC: >256 µg/mL), **5** (MIC: >256 µg/mL), and **6** (MIC: >256 µg/mL)—culminating in poor TI values. While hydrophobicity is crucial, excessive hydrophobicity, as observed in peptides **2**–**6**, likely results in aggregate formation or sequestration within the hydrophobic matrix of *A. baumannii* biofilms, ultimately reducing antibacterial efficacy. These findings emphasise that increasing hydrophobicity, without considering other key physicochemical parameters, is insufficient to improve the safety profile of gramicidin S.

Our findings suggest that **7** possesses an optimal balance between hydrophobicity and cationicity, which likely act synergistically to facilitate penetration of *A. baumannii* biofilms, while significantly reducing cytotoxicity, even at concentrations eight times higher than those required for complete bacterial eradication (Figure S1). Peptides **1** and **7** not only demonstrate potent activity against *A. baumannii* but also retain broad-spectrum efficacy, with **1** (MIC: 2 µg/mL) exhibiting a two-fold improvement in bactericidal potency over gramicidin S (MIC: 4 µg/mL) against both MSSA and MRSA clinical isolates.

**Figure 2:**
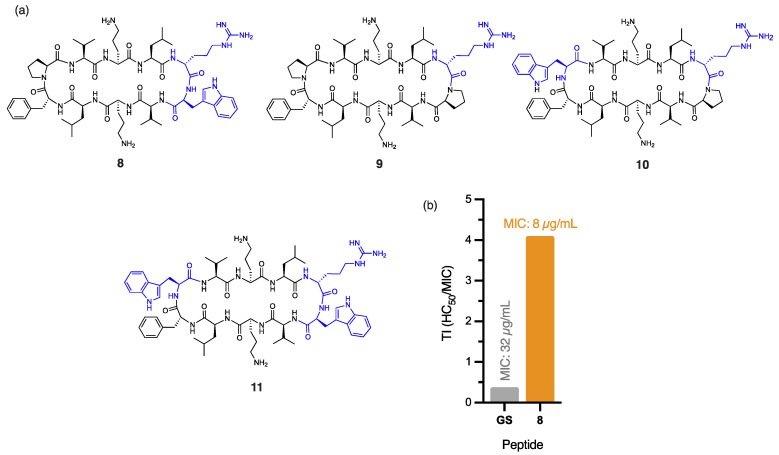
(**a**) Structure of peptides **8**–**11**, with residues highlighted in blue indicating substitutions relative to the parent compound. (**b**) Calculated therapeutic indices (TIs) for peptide **8** and gramicidin S against *E. coli*.

The outer membrane of *E. coli* poses a substantial barrier to peptide penetration due to the unique composition of its lipopolysaccharides, with O-antigen side chains and specific core oligosaccharides effective at repelling poorly cationic or overly hydrophobic peptides [24]. Peptide **8**, with a single ^D^Arg/Trp in place of the ^D^Phe/Pro β-turn of gramicidin S (Figure 2a), displayed a significant four-fold improvement in antibacterial activity (MIC: 8 µg/mL) against *E. coli*, compared to gramicidin S (MIC: 32 µg/mL). The addition of cationic ^D^Arg within the β-turn region of **8** likely promotes interaction with the lipopolysaccharide layer through bidentate hydrogen bonding, while the hydrophobic Trp improves the anchoring and penetration of the outer bacterial membrane [25]. Trp is known to interact favourably with bacterial membranes by aligning at the lipid bilayer interface. Its amphipathic nature allows the hydrophobic benzene ring to integrate into the membrane core, while the polar pyrrole NH group stabilises interactions through hydrogen bonding with phospholipid head groups [26]. Thus, we postulate that the aromatic Trp side chain of peptide **8** promotes stronger lipid spreading and hence, more pronounced membrane effects than those of gramicidin S [16]. This dual functionality strengthens peptide–membrane interactions, establishing Trp as a prevalent feature in many membrane-targeting antimicrobial peptides [25]. Furthermore, the guanidinium side chain of Arg provides a complementary function that enables strong electrostatic interactions with the negatively charged bacterial surface. This property likely enhances initial binding affinity and can also facilitate deeper membrane penetration, supporting peptide translocation and improved antimicrobial efficacy of peptide **8**. The crucial role of cationic ^D^Arg for antibacterial potency against *E. coli*, unlike for *A. baumannii*, is exemplified by the stark contrast in MIC values for peptides **8** (8 µg/mL) and **2** (256 µg/mL), where the only structural difference is the replacement of ^D^Arg with ^D^Phe in **2** (Figure 1a and Figure 2a). Interestingly, the close proximity between the Trp and ^D^Arg residues in **8** enhances activity, as peptides **9** (no Trp), **10** (distal Trp), and **11** (dual Trp) (Figure 2a) each exhibited weaker antibacterial activity (16 µg/mL). The significant reduction in haemolytic toxicity (HC_50_: 32.81 ± 0.51 µg/mL) for peptide **8** contributes to its markedly improved therapeutic index (4.10), which is more than ten times that of gramicidin S (0.38), thereby overcoming a key barrier in antibiotic development. Notably, **8** also exhibits high potency (5 µg/mL) against methicillin-resistant (ATCC USA300) *S. aureus* [10], comparable to gramicidin S (4 µg/mL). The broad-spectrum efficacy of this key derivative against both Gram-negative and Gram-positive pathogens establishes it as a powerful contender in the fight against multidrug-resistant bacteria.

**Figure 3:**
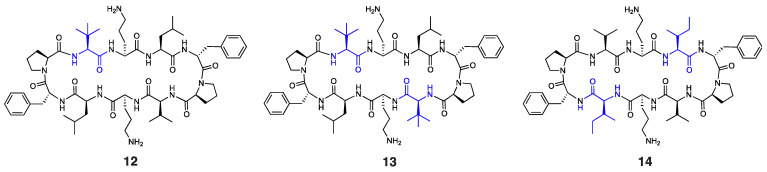
Structure of peptides **12**–**14**, with residues highlighted in blue indicating substitutions relative to the parent compound.

It is worth noting that other Trp-containing analogues that lack ^D^Arg in the β-turn region, namely, **2** (1 × Trp), **3** (2 × proximal Trp), **4** (2 × distal Trp), **5** (3 × Trp), and **6** (4 × Trp) (Figure 1a), showed no bactericidal activity at the highest concentration tested (MIC: >256 µg/mL). Substituting Val and/or Leu residues in gramicidin S with *tert*-leucine (Tle), as in peptides **1**, **12**, and **13** (Figure 1a and Figure 3), had no impact on the potency against *E. coli*. Moreover, substituting both leucine residues with isoleucine, as in peptide **14** (Figure 3), resulted in a significant reduction in antibacterial activity (MIC: 256 µg/mL), despite being a subtle modification.

**Figure 4:**
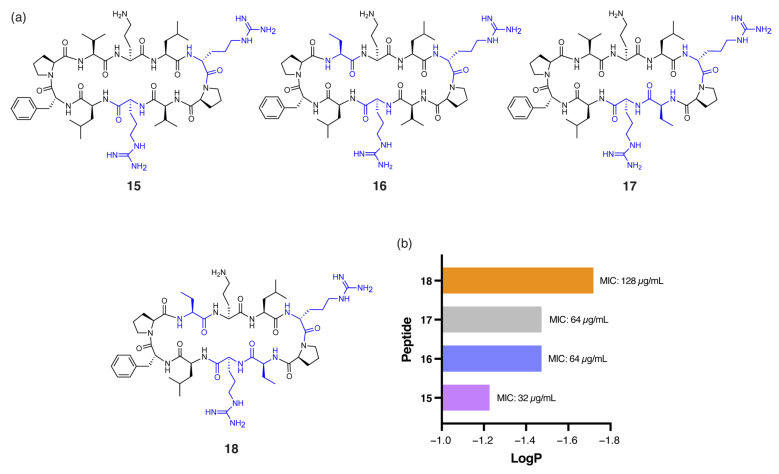
(**a**) Structure of peptides **15**–**18**, with residues highlighted in blue indicating substitutions relative to the parent compound. (**b**) Correlation between calculated logP of peptides **14**–**17** and experimental MIC values against *P. aeruginosa*.

*Pseudomonas aeruginosa* (*P. aeruginosa*) is particularly challenging to target due to its highly impermeable outer membrane and robust efflux mechanisms [27]. Among the peptides tested, **7** demonstrated notably enhanced antibacterial activity against *P. aeruginosa* (MIC: 32 µg/mL) compared to gramicidin S (128 µg/mL). The single ^D^Arg residue within the β-turn region, combined with four Tle residues within the β-strands, reduces the hydrophobicity of **7** (t_R_ = 16.92 min) compared to gramicidin S (t_R_ = 18.57 min). Similarly, peptide **15** (Figure 4a), with ^D^Arg positioned in both the β-turn and β-strand, was equipotent to **7**. However, further reduction in global hydrophobicity led to progressively diminished antibacterial activity, as demonstrated by peptides **16**, **17**, and **18** (Figure 4a). These analogues have either one or both Val residues replaced with the less hydrophobic α-aminobutyric acid (Abu) and exhibited lower RP-HPLC retention times (**16**: t_R_ = 16.24, **17**: t_R_ = 16.32 min, **18**: t_R_ = 16.03 min) and reduced predicted logP values (Figure 4b) compared to **15** (t_R_ = 16.47 min). Peptides **16** and **17**, each with one Abu residue, exhibited an MIC of 64 µg/mL, while the more polar **18**, with two Abu residues, showed substantially reduced activity (MIC: 128 µg/mL). This excessive hydrophilicity may promote the sequestration of the peptides within the lipopolysaccharide (LPS) layer, preventing effective penetration of the inner bacterial membrane [9].

**Figure 5:**
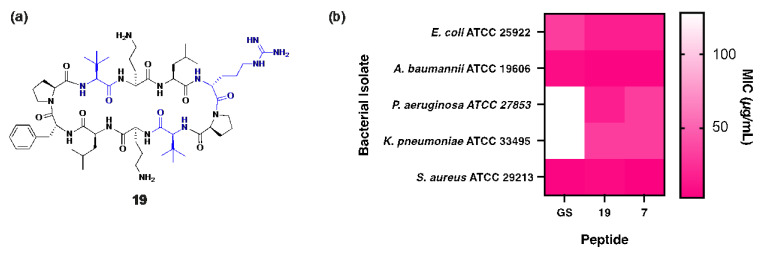
(**a**) Structure of peptide **19**, with residues highlighted in blue indicating substitutions relative to the parent compound. (**b**) Heat map showing comparison of MIC values for peptides **7**, **19** and gramicidin S against each bacterial isolate.

Peptide **19** shares structural similarity with **7** but incorporates two Tle and two Leu residues within the β-strand regions (Figure 5a) to slightly increase global hydrophobicity (**19**: t_R_ = 17.15 min), optimally positioning the peptide between gramicidin S (t_R_ = 18.57 min) and peptide **7** (t_R_ = 16.92 min). Unsurprisingly, superior antibacterial activity against *P. aeruginosa* was observed for **19** (MIC: 16 µg/mL), representing a substantial 8-fold improvement over gramicidin S (MIC: 128 µg/mL), along with an 11-fold increase in the therapeutic index. Notably, peptide **19** also demonstrated enhanced bactericidal potency (MIC: 3 µg/mL) against both MSSA and MRSA clinical isolates [10] compared to gramicidin S (MIC: 4 µg/mL), highlighting its broad-spectrum efficacy (Figure 5b).

*Klebsiella pneumoniae* (*K. pneumoniae*) is an opportunistic pathogen renowned for its thick capsular polysaccharide layer (CPS), a key virulence factor that facilitates immune evasion and resistance to phagocytosis [28]. With the CPS reaching a thickness of up to 400 nm [28], this structural feature presents a significant barrier to peptide permeation, necessitating an optimal balance of key physicochemical parameters for efficient translocation. The capsule, composed of extracellular polysaccharides, acts as a potent defence mechanism by masking the bacteria from immune recognition and neutralising innate host defence peptides, including last-resort polymyxin antibiotics [29]. The anionic nature of the capsule promotes electrostatic interactions with cationic antimicrobial peptides, while hydrophobic elements facilitate capsule binding through non-ionic interactions [30]. However, an overly strong affinity to the capsule may trap antimicrobial peptides, preventing them from reaching their bacterial membrane targets and thereby reducing their effectiveness [29].

In this context, peptide **9**, which contains ^D^Arg, and peptides **8** and **11**, which include both ^D^Arg and Trp (Figure 2), were found to be most effective against *K. pneumoniae* (MIC: 16 µg/mL), demonstrating an eight-fold greater potency compared to gramicidin S (MIC: 128 µg/mL). Notably, the enhanced antibacterial activity and low haemolytic toxicity of **9** culminated in a significant 25-fold improvement (2.45) to the TI of gramicidin S (0.096). It is likely that the cationic guanidinium of ^D^Arg and the extensive π-electron system of hydrophobic Trp both facilitate interactions with the interfacial region of the bacterial membrane and assist in bypassing the capsule barrier [31]. The presence of a Trp residue proximal to ^D^Arg, as in **8** and **11**, enhances potency compared to the distal arrangement in **10** (MIC: 32 µg/mL), likely due to changes in the amphiphilic moment that modify the electronic properties of the peptide to overcome the capsule-mediated sequestration that typically inhibits peptide activity [29].

Consistent with our results obtained for *E. coli* and *P. aeruginosa*, all other Trp-containing peptides that lack ^D^Arg within the β-turn region (**2**–**6**) exhibited no antibacterial activity against *K. pneumoniae* within the tested range (MIC: >256 µg/mL), highlighting the critical contribution of this cationic residue within the β-turn region. The absence of ^D^Arg clearly compromises the ability of these peptides to effectively target *K. pneumoniae*, reinforcing the notion that a strategic balance of cationicity and hydrophobicity is crucial for overcoming the capsule barrier. These findings underscore the interplay between ^D^Arg and Trp, suggesting their combined presence within the β-turn region is essential for exerting antibacterial effects, particularly against *E. coli* and *K. pneumoniae*.

To further evaluate the therapeutic potential of the most effective analogues, an in vitro nephrotoxicity assay (MTT) was conducted using human embryonic kidney (HEK-293) cells [10]. Each peptide—**7** (12.60 µg/mL), **8** (13.62 µg/mL), **9** (18.66 µg/mL), and **19** (9.75 µg/mL)—showed reduced nephrotoxic effects compared to gramicidin S (6.45 µg/mL), highlighting their improved safety profile and potential for further development.

Collectively, our findings underscore the importance of tailoring peptide design based on the unique structural barriers presented by each Gram-negative ESKAPE pathogen. Cationicity, primarily conferred by the incorporation of ^D^Arg, was found to be crucial for reducing haemolytic toxicity toward human red blood cells while also enhancing antibacterial potency against all tested bacterial strains, except *A. baumannii*, suggesting that they may share common resistance mechanisms. While the highly negatively charged lipopolysaccharide (LPS) layer and thick capsular polysaccharide (CPS) of each bacterial species likely facilitate interactions with cationic/hydrophobic peptides, the robust hydrophobic biofilm of *A. baumannii* appears to repel peptides with excessive cationicity. Hence, while hydrophobicity was essential for targeting *A. baumannii*, this characteristic alone was insufficient to achieve potent antibacterial activity against the other species, likely due to aggregation or sequestration within the bacterial capsule/membranes. These species-specific differences underscore the need for a nuanced approach to peptide design, balancing cationic and hydrophobic properties to ensure broad-spectrum efficacy while minimising cytotoxicity.

## 3. Materials and Methods

### 3.1. Chemicals

All reagents and solvents were purchased from Chem-Impex International (Wood Dale, IL, USA), Merck (Bayswater, Australia) and Enamine Ltd. (Kyiv, Ukraine).

### 3.2. Peptide Synthesis

Gramicidin S and analogues **1**–**19** were synthesised using standard Fmoc-based solid-phase peptide synthesis (SPPS) [32]. Fmoc-^D^Phe-OH (0.8 mmol/g, 5 equiv.) or Fmoc-^D^Trp-OH (0.8 mmol/g, 5 equiv., peptide **6**) was loaded onto 2-chlorotrityl resin by treatment with DIPEA (10 equiv.) in DCM:DMF (9:1) overnight. Unoccupied sites were capped using DCM/MeOH/DIPEA (17:2:1) (2 × 15 min). The Fmoc protecting group was removed by treating the resin with 20% piperidine in DMF (15 min). Coupling of each Fmoc-protected amino acid (5 equiv.) was achieved using DIPEA (10 equiv.) and HATU (5 equiv., 0.5 M) in DMF (20 mL/mmol, 1 h). The completion of amino acid coupling was assessed using the TNBS test—if a positive result was observed, indicating incomplete coupling, the reaction was repeated under the same conditions to ensure complete attachment of the residue. The peptide chain was sequentially elongated until the desired linear sequence was completed. Cleavage of the linear peptides from the resin was carried out using a cleavage cocktail consisting of 2% TFA, 93% DCM, 2.5% DODT, and 2.5% TIPS (10 mL/0.1 mmol, 2 h). The cleavage mixture was evaporated under a nitrogen stream to a final volume of 0.5–1 mL. The peptides were then precipitated in diethyl ether (20 mL), cooled in an acetone/dry ice bath for 10 min, and centrifuged (5700 rpm, 10 min). The supernatant was discarded, and the precipitate was dried under a nitrogen stream before being redissolved in 20% ACN/H_2_O and lyophilised to obtain the linear peptides as white solids. For cyclisation, the linear peptides were dissolved in anhydrous DMF (150 mL/mmol) containing diphenylphosphoryl azide (DPPA, 3 equiv.) and DIPEA (6 equiv.), and the reaction was allowed to proceed overnight at room temperature under a nitrogen atmosphere. The reaction mixture was concentrated to 0.5–1 mL under a nitrogen stream, and the cyclic peptides were precipitated in diethyl ether (20 mL), cooled in an acetone/dry ice bath, and centrifuged (5700 rpm, 10 min). The precipitate was collected, dried under a nitrogen stream, redissolved in 20% ACN/H_2_O, and lyophilised to yield cyclic peptides as white solids. Final deprotection of Boc and Pbf protecting groups was achieved by treating the peptides with 95% TFA, 2.5% TIPS, and 2.5% H_2_O. The solvent was evaporated under nitrogen to yield the final peptides.

### 3.3. Reversed-Phase High-Performance Liquid Chromatography (RP-HPLC)

Peptide purification was carried out via preparative RP-HPLC on an Agilent 1260 Infinity II system (Santa Clara, CA, USA) equipped with a 21.2 × 150 mm Agilent ZORBAX Eclipse Plus C_18_ column, operating at a flow rate of 25 mL/min. The mobile phase consisted of acetonitrile containing 0.0008% trifluoroacetic acid (*v*/*v*) as the organic phase and water with 0.001% TFA as the aqueous phase. Following purification, peptides **1**–**19** were lyophilised and obtained as white powders. Analytical HPLC analysis was performed on a Hewlett-Packard Series 1100 instrument using a Phenomenex (Torrance, CA, USA) Kinetex C_18_ column (150 mm × 4.6 mm, 2.6 µm particle size), under identical solvent conditions, to confirm purity. All peptides were >95% pure.

### 3.4. High-Resolution Mass Spectrometry (HRMS)

High-resolution mass spectrometry was conducted using a Thermo Scientific LTQ Orbitrap XL ETD system (Waltham, MA, USA) coupled with an Ultimate 3000 RSL HPLC for sample introduction via flow injection. Samples were infused at a rate of 5 μL/min, and ionisation was achieved through electrospray (ESI) in positive mode. Spectral data were collected across an *m*/*z* range of 100 to 1600 and subsequently processed using the XCalibur software suite (version 4.2, Thermo Scientific, Waltham, MA, USA).

### 3.5. Antibacterial Assays (MIC Determination)

The antibacterial efficacy of peptides was assessed using the broth microdilution method in accordance with Clinical and Laboratory Standards Institute (CLSI) guidelines (M07-A10, 2015 [33]). The minimum inhibitory concentrations (MICs) were determined against *Escherichia coli* (ATCC 25922), *Klebsiella pneumoniae* (ATCC 33495), *Pseudomonas aeruginosa* (ATCC 27853), *Acinetobacter baumannii* (ATCC 19606), and *Staphylococcus aureus* (ATCC 29213 and ATCC USA300). Bacterial suspensions were standardised to 5 × 10^4^ colony-forming units (CFUs)/well in cation-adjusted Mueller–Hinton broth (CAMHB). Peptides were dissolved in 25% DMSO and serially diluted two-fold across 96-well plates, reaching final concentrations ranging from 256 to 0.125 µg/mL. The wells were inoculated, ensuring a final volume of 100 µL per well, with a DMSO concentration of 1% (*v*/*v*) to minimise solvent interference. Plates were incubated at 37 °C for 16–20 h, and the MIC was recorded as the lowest peptide concentration that completely inhibited visible bacterial growth. Vancomycin was used as the positive control for S. aureus. Polymyxin B served as the positive control for Gram-negative bacteria. CAMHB with 1% (*v*/*v*) DMSO (without peptides) was used as the negative control. All experiments were performed in triplicate (*n* = 3). Bacterial isolates were stored in tryptone soy broth with 30% glycerol at −80 °C for long-term preservation.

### 3.6. Haemolysis Assays

To evaluate haemolytic activity, fresh human blood was collected from three unrelated healthy volunteers to ensure donor variability. Red blood cells (RBCs) were separated by centrifugation at 3000 rpm for 5 min at 4 °C, followed by washing in HEPES-buffered saline (HBS; 150 mM NaCl, 5 mM HEPES-NaOH, pH 7.4). The samples were pooled, mixed, and all experiments were performed in triplicate to enhance reproducibility. Blood samples were used within 48 h of collection to preserve erythrocyte integrity. A 1% RBC suspension in HBS (200 μL) was distributed into 96-well plates, and peptide solutions (100 μL) were added to reach final concentrations ranging from 0.125 to 256 μg/mL. After a 1 h incubation at 37 °C, the plates were centrifuged (3000 rpm, 5 min, 4 °C), and 80 μL of each supernatant was transferred to a fresh microplate. Absorbance readings were taken at 540 nm and 410 nm to assess protein content and haemoglobin release, respectively. HBS served as a negative control and 1% Triton X-100 as a positive control (*n* = 3).

### 3.7. MTT Assay

The cytotoxic potential of the peptides toward human kidney cells was assessed using an MTT-based viability assay, adapted from previously described protocols [9]. Human embryonic kidney (HEK-293) cells, kindly provided by Dr. Samuel Evans (Neuroimmunopharmacology Laboratory, University of Adelaide), were seeded into 96-well plates at a density of 1 × 10^4^ cells per well in 280 μL of high-glucose DMEM supplemented with 10% fetal bovine serum (FBS), 2 mM L-glutamine, and 1% penicillin–streptomycin. Plates were incubated at 37 °C with 5% CO_2_ for 24 h. Following cell attachment, serial dilutions of gramicidin S and peptides 1–19 were applied (final concentrations: 256 to 0.125 μg/mL; *n* = 3), and the cells were incubated for an additional 24 h under identical conditions. After treatment, the media were removed and cells were gently washed with PBS. MTT reagent (100 μL of a 0.25 mg/mL solution in serum-free DMEM) was then added to each well, followed by a 4 h incubation at 37 °C. Subsequently, the supernatant was discarded, and the formed formazan crystals were solubilised by adding 100 μL of DMSO. Plates were gently agitated for 10 min before measuring absorbance at 570 nm to quantify cell viability.

## 4. Conclusions

This study demonstrates strategic structural modifications to the FDA-approved antibiotic gramicidin S that expand its antibacterial spectrum to effectively target the Gram-negative pathogens *E. coli*, *K. pneumoniae*, *P. aeruginosa*, and *A. baumannii*, in addition to Gram-positive *S. aureus*. The incorporation of ^D^Arg, coupled with the strategic optimisation of hydrophobicity through *tert*-leucine (Tle) and tryptophan (Trp), effectively overcomes the critical structural barriers unique to each bacterial species. Peptide **8** exhibited a four-fold improvement in antibacterial activity against *E. coli* (MIC: 8 µg/mL) compared to gramicidin S (32 µg/mL) while achieving a ten-fold increase in the therapeutic index. Against *K. pneumoniae*, peptides **8, 9**, and **11** (MIC: 16 µg/mL) demonstrated superior efficacy, significantly outperforming gramicidin S (MIC: 128 µg/mL), with **9** improving the therapeutic index by a substantial 25-fold. Peptide **19** achieved a notable 8-fold enhancement in potency (MIC: 16 µg/mL) against *P. aeruginosa*, while **7**, comprising four non-proteinogenic Tle residues within the β-strand regions, and cationic ^D^Arg within a single β-turn, demonstrated a remarkable 27-fold enhancement in the therapeutic index. Similarly, against *A. baumannii*, peptide **7** achieved a two-fold increase in activity (MIC: 4 µg/mL) combined with a marked reduction in haemolytic toxicity. Collectively, these results provide critical insights into key strategies for the rational design of peptides to overcome the unique species-specific structural barriers, establishing a robust framework for developing innovative therapeutic candidates to address the escalating global challenge of multidrug-resistant Gram-negative bacterial infections.

## Figures and Tables

**Table 1:** MIC values and haemolytic toxicity (HC_50_) towards human red blood cells for gramicidin S and peptides **1**–**19**.

Peptide	MIC (µg/mL)						HC_50_ (µg/mL)
	*S. aureus* ATCC 29213	*S. aureus* ATCC USA300	*E. coli* ATCC 25922	*P. aeruginosa* ATCC 27853	*K. pneumonia* ATCC 33495	*A. baumannii* ATCC 19606	
GS	4	4	32	128	128	8	12.34 ± 9.27
**1**	2	2	32	64	128	4	5.90 ± 0.23
**2**	4	4	256	>256	>256	64	9.04 ± 0.16
**3**	8	8	>256	>256	>256	128	5.85 ± 0.11
**4**	8	8	>256	>256	>256	>256	12.24 ± 0.47
**5**	64	64	>256	>256	>256	>256	37.21 ± 1.70
**6**	32	32	>256	>256	>256	>256	55.58 ± 1.81
**7**	6	6	16	32	32	4	84.09 ± 1.02
**8**	5	5	8	32	16	8	32.81 ± 0.51
**9**	8	8	16	32	16	8	39.21 ± 0.46
**10**	5	5	16	32	32	8	31.88 ± 0.47
**11**	5	5	16	32	16	8	19.64 ± 0.37
**12**	4	4	32	128	128	8	7.49 ± 0.35
**13**	4	4	32	128	256	32	6.26 ± 0.35
**14**	4	4	256	>256	>256	>256	8.11 ± 0.24
**15**	8	8	16	32	16	8	33.82 ± 1.11
**16**	16	16	32	64	32	16	65.32 ± 1.72
**17**	16	16	32	64	32	16	70.52 ± 1.93
**18**	32	32	64	128	64	32	121.6 ± 4.49
**19**	3	3	16	16	32	4	17.70 ± 0.52

## Data Availability

The original contributions presented in this study are included in the article and Supplementary Materials. Further inquiries can be directed to the corresponding author.

## Supplementary Materials

The following supporting information can be downloaded at: https://www.mdpi.com/article/10.3390/antibiotics14050423/s1, Figure S1: Normalised haemolysis (%) of human red blood cells in response to increasing concentrations of gramicidin S and peptides **1**–**19**; Figure S2. HRMS of gramicidin S. [M + H]^+^ calc’d = 1141.7139, [M + H]^+^ found = 1141.7134; Figure S3. HRMS of peptide **1**. [M + H]^+^ calc’d = 1169.7450, [M + H]^+^ found = 1169.7451; Figure S4. HRMS of peptide **2**. [M + H]^+^ calc’d = 1230.7403, [M + H]^+^ found = 1230.7404; Figure S5. HRMS of peptide **3**. [M + H]^+^ calc’d = 1269.7512, [M + H]^+^ found = 1269.7501; Figure S6. HRMS of peptide **4**. [M + H]^+^ calc’d = 1319.7668, [M + H]^+^ found = 1319.7650; Figure S7. HRMS of peptide **5**. [M + H]^+^ calc’d = 1358.7777, [M + H]^+^ found = 1358.7774; Figure S8. HRMS of peptide **6**. [M + 2H]^+^ calc’d = 1398.7964, [M + 2H]^+^ found = 1398.7955; Figure S9. HRMS of peptide **7**. [M + H]^+^ calc’d = 1178.7777, [M + H]^+^ found = 1178.7779; Figure S10. HRMS of peptide **8**. [M + H]^+^ calc’d = 1239.7730, [M + H]^+^ found = 1239.7740; Figure S11. HRMS of peptide **9**. [M + H]^+^ calc’d = 1150.7464, [M + H]^+^ found = 1150.7457; Figure S12. HRMS of peptide **10**. [M + H]^+^ calc’d = 1239.7730, [M + H]^+^ found = 1239.7734; Figure S13. HRMS of peptide **11**. [M + H]^+^ calc’d = 1328.7995, [M + H]^+^ found = 1328.7989; Figure S14. HRMS of peptide **12**. [M + H]^+^ calc’d = 1155.7294, [M + H]^+^ found = 1155.7292; Figure S15. HRMS of peptide **13**. [M + H]^+^ calc’d = 1169.7450, [M + H]^+^ found = 1169.7450; Figure S16. HRMS of peptide **14**. [M + 2H]^+^ calc’d = 1142.7215, [M + 2H]^+^ found = 1142.7132; Figure S17. HRMS of peptide **15**. [M + H]+ calc’d = 1192.7682, [M + H]^+^ found = 1192.7684; Figure S18. HRMS of peptide **16**. [M + H]^+^ calc’d = 1178.7526, [M + H]^+^ found = 1178.7525; Figure S19. HRMS of peptide **17**. [M + H]^+^ calc’d = 1178.7526, [M + H]^+^ found = 1178.7519; Figure S20. HRMS of peptide **18**. [M + H]^+^ calc’d = 1164.7369, [M + H]^+^ found = 1164.7371; Figure S21. HRMS of peptide **19**. [M + H]^+^ calc’d = 1178.7777, [M + H]^+^ found = 1178.7771; Figure S22. Analytical RP-HPLC traces for gramicidin S and peptides **1**–**19**; Table S1. Primary structures of gramicidin S and peptides **1**–**19**; Table S2. HRMS data for gramicidin S and peptides **1**–**19**; Table S3. Analytical RP-HPLC for gramicidin S and peptides **1**–**19** was performed on an HP Series 1100 with a Phenomenex Kinetex 2.6 μM C18 column (150 × 4.60 mm) at a flow rate of 0.7 mLmin^−1^ using ACN/TFA (100:0.0008 *v*/*v*) and water/TFA (100:0.001 *v*/*v*) as organic and aqueous buffers, respectively.

## Abbreviations

The following abbreviations are used in this manuscript:

Abu	α-aminobutyric acid
ACN	Acetonitrile
CAMHB	Cation-adjusted Mueller-Hinton broth
CLSI	Clinical and Laboratory Standards Institute
CPS	Capsular polysaccharide
^D^Arg	D-arginine
DCM	Dichloromethane
DIPEA	N,N-diisopropylethylamine
DMF	Dimethylformamide
DMSO	Dimethyl sulfoxide
DPhe	D-phenylalanine
DPPA	Diphenylphosphoryl azide
^D^Trp	D-tryptophan
Fmoc	Fluorenylmethyloxycarbonyl
GS	Gramicidin S
HATU	O-(7-azabenzotriazol-1-yl)-N,N,N′,N′-tetramethyluronium hexafluorophosphate
HC_50_	50% haemolytic concentration
HEK-293	Human embryonic kidney 293 cells
HEPES	4-(2-hydroxyethyl)-1-piperazineethanesulfonic acid
HBS	HEPES-buffered saline
HRMS	High-resolution mass spectrometry
logP	Logarithm of the partition coefficient
LPS	Lipopolysaccharide
MIC	Minimum inhibitory concentration
MRSA	Methicillin-resistant Staphylococcus aureus
MSSA	Methicillin-sensitive Staphylococcus aureus
Pbf	2,2,4,6,7-pentamethyldihydrobenzofuran-5-sulfonyl
RP-HPLC	Reverse-phase high-performance liquid chromatography
SPPS	Solid-phase peptide synthesis
TFA	Trifluoroacetic acid
THF	Tetrahydrofuran
TI	Therapeutic index
TIPS	Triisopropylsilane
Tle	Tert-leucine
Trp	Tryptophan
t_R_	Retention time
