# Supplementary material for: Broad-Spectrum Gramicidin S Derivatives with Potent Activity Against Multidrug-Resistant Gram-Negative ESKAPE Pathogens
Source: Antibiotics (Basel). 2025 Apr 22;14(5):423. doi: 10.3390/antibiotics14050423 (PMC12108418; doi:10.3390/antibiotics14050423)
Supplement: Supplementary file 1 [file antibiotics-14-00423-s001.zip › antibiotics-3606003-supplementary.pdf]

Supplementary Materials:

Broad-Spectrum Gramicidin S Derivatives with Potent Activity Against Multidrug-Resistant Gram-Negative ESKAPE Pathogens

John T. Kalyvas[1], Yifei Wang[1], Ornella Romeo[2], John R. Horsley[1], and Andrew D. Abell[1*]

1 Department of Chemistry, School of Physics, Chemistry and Earth Sciences, The University of Adelaide, Ade-laide, SA 5005, Australia; john.kalyvas@adelaide.edu.au (J.T.K.); yifei.wang@adelaide.edu.au (Y.W.); john.horsley@adelaide.edu.au (J.R.H.)

2 Research Centre for Infectious Diseases, School of Biological Sciences, The University of Adelaide,

Adelaide, SA 5005, Australia; ornella.romeo@adelaide.edu.au

* Correspondence: andrew.abell@adelaide.edu.au

Table of Contents:

1. **Peptide Primary Sequences** S3
    - 1.1. Primary structures of gramicidin S and peptides **1 - 19** S3
2. **Biological Assays** S4
    - 2.1. Haemolysis curves for gramicidin S and peptides **1 - 19** S4
3. **Mass Spectrometry** S7
4. **RP-HPLC Traces** S18

1. Peptide Primary Sequences

1.1 Primary structures of gramicidin S and peptides 1-19

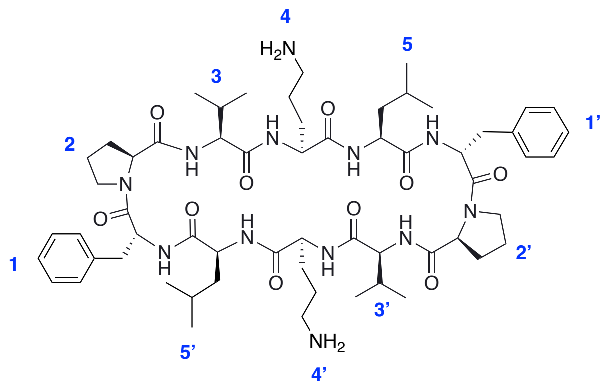

Gramicidin S

Table S1. Primary structures of gramicidin S and peptides **1-19**.

	**Residue**									
**Peptide**	**1**	**1'**	**2**	**2'**	**3**	**3'**	**4**	**4'**	**5**	**5'**
**GS**	^{D}Phe	^{D}Phe	Pro	Pro	Val	Val	Orn	Orn	Leu	Leu
**1**	^{D}Phe	^{D}Phe	Pro	Pro	Tle	Tle	Orn	Orn	Tle	Tle
**2**	^{D}Phe	^{D}Phe	Trp	Pro	Val	Val	Orn	Orn	Leu	Leu
**3**	^{D}Trp	^{D}Phe	Trp	Pro	Val	Val	Orn	Orn	Leu	Leu
**4**	^{D}Phe	^{D}Phe	Trp	Trp	Val	Val	Orn	Orn	Leu	Leu
**5**	^{D}Trp	^{D}Phe	Trp	Trp	Val	Val	Orn	Orn	Leu	Leu
**6**	^{D}Trp	^{D}Trp	Trp	Trp	Val	Val	Orn	Orn	Leu	Leu
**7**	^{D}Phe	^{D}Arg	Pro	Pro	Tle	Tle	Orn	Orn	Tle	Tle
**8**	^{D}Arg	^{D}Phe	Trp	Pro	Val	Val	Orn	Orn	Leu	Leu
**9**	^{D}Arg	^{D}Phe	Pro	Pro	Val	Val	Orn	Orn	Leu	Leu
**10**	^{D}Phe	^{D}Arg	Trp	Pro	Val	Val	Orn	Orn	Leu	Leu
**11**	^{D}Phe	^{D}Arg	Trp	Trp	Val	Val	Orn	Orn	Leu	Leu
**12**	^{D}Phe	^{D}Phe	Pro	Pro	Tle	Val	Orn	Orn	Leu	Leu
**13**	^{D}Phe	^{D}Phe	Pro	Pro	Tle	Tle	Orn	Orn	Leu	Leu
**14**	^{D}Phe	^{D}Phe	Pro	Pro	Val	Val	Orn	Orn	Ile	Ile
**15**	^{D}Arg	^{D}Phe	Pro	Pro	Val	Val	Arg	Orn	Leu	Leu
**16**	^{D}Arg	^{D}Phe	Pro	Pro	Val	Abu	Arg	Orn	Leu	Leu
**17**	^{D}Arg	^{D}Phe	Pro	Pro	Abu	Val	Arg	Orn	Leu	Leu
**18**	^{D}Arg	^{D}Phe	Pro	Pro	Abu	Abu	Arg	Orn	Leu	Leu
**19**	^{D}Phe	^{D}Arg	Pro	Pro	Tle	Tle	Orn	Orn	Leu	Leu

2. Biological Assays

2.1 Haemolysis curves for gramicidin S and peptides 1 – 19 against human red blood cells

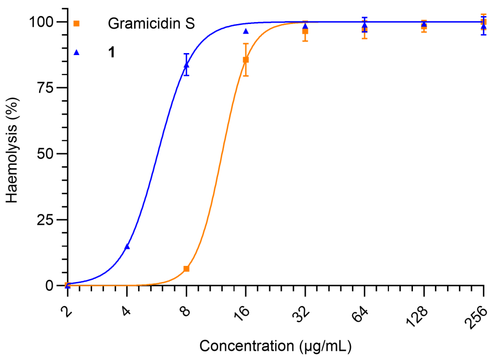

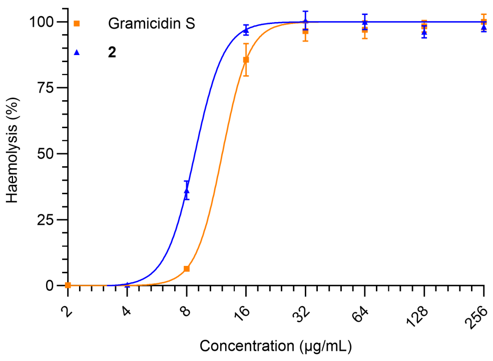

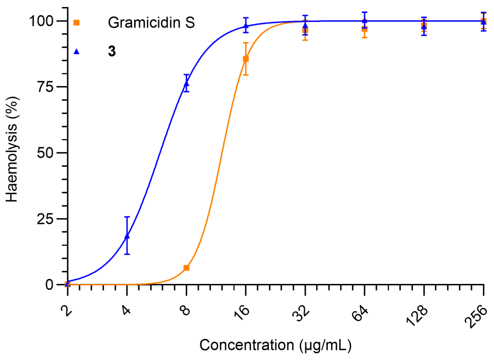

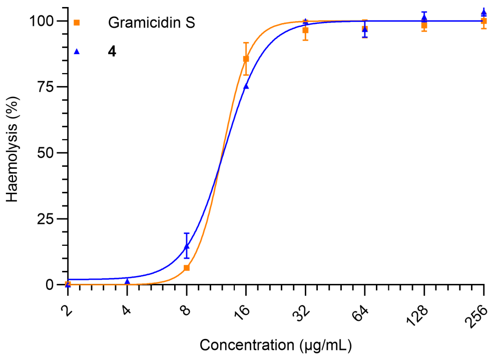

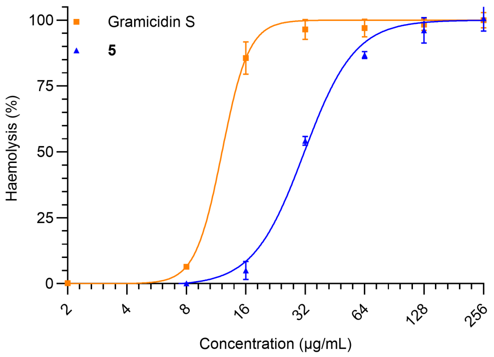

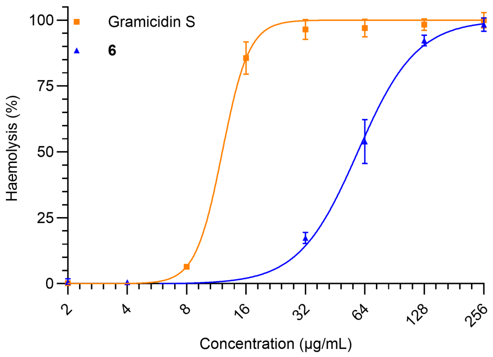

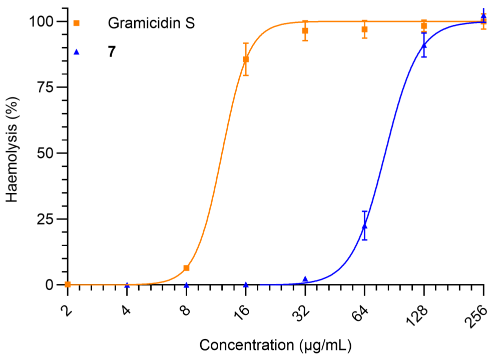

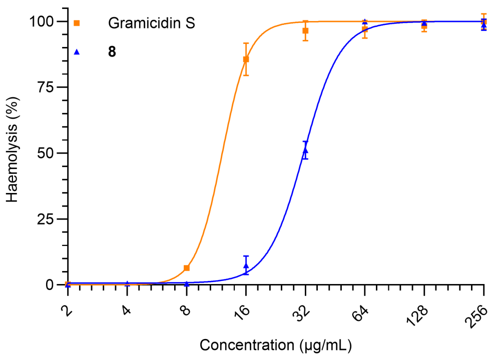

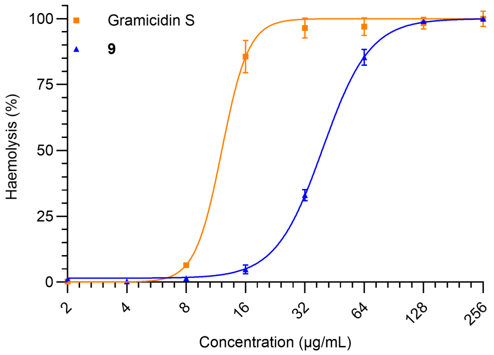
Gramicidin S
9
Haemolysis (%)
100
75
50
25
0
2
4
8
16
32
64
128
256
Concentration (μg/mL)

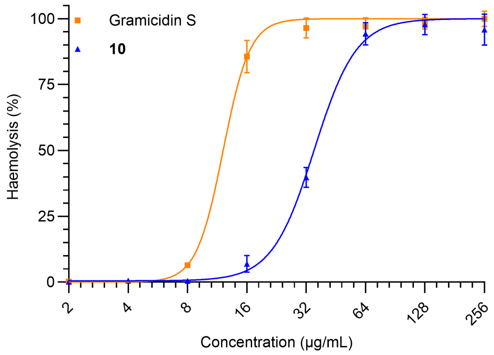
Gramicidin S
10
Haemolysis (%)
100
75
50
25
0
2
4
8
16
32
64
128
256
Concentration (μg/mL)

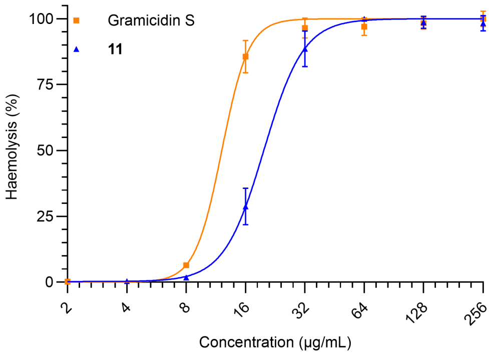
Gramicidin S
11
Haemolysis (%)
100
75
50
25
0
2
4
8
16
32
64
128
256
Concentration (μg/mL)

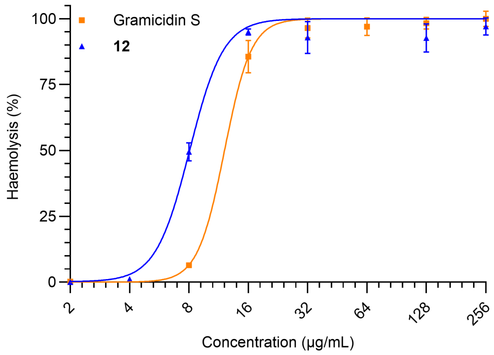
Gramicidin S
12
Haemolysis (%)
100
75
50
25
0
2
4
8
16
32
64
128
256
Concentration (μg/mL)

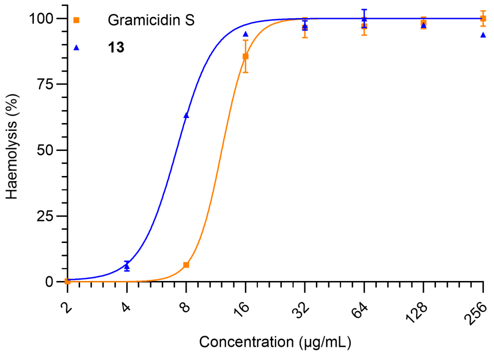
Gramicidin S
13
Haemolysis (%)
100
75
50
25
0
2
4
8
16
32
64
128
256
Concentration (μg/mL)

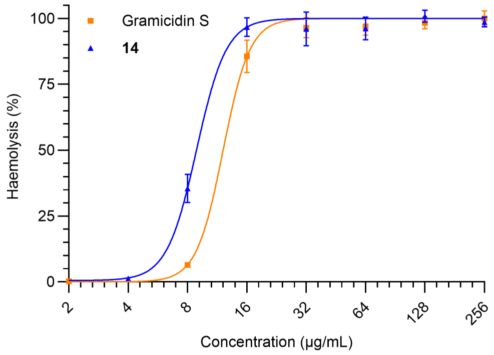
Gramicidin S
14
Haemolysis (%)
100
75
50
25
0
2
4
8
16
32
64
128
256
Concentration (μg/mL)

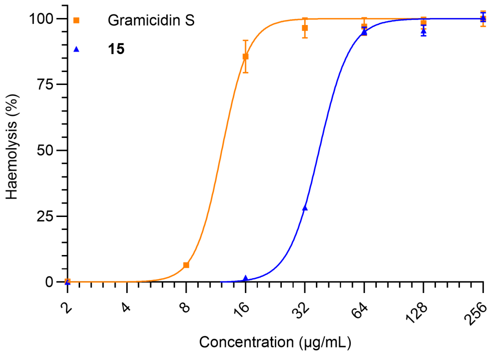
Gramicidin S
15
Haemolysis (%)
100
75
50
25
0
2
4
8
16
32
64
128
256
Concentration (μg/mL)

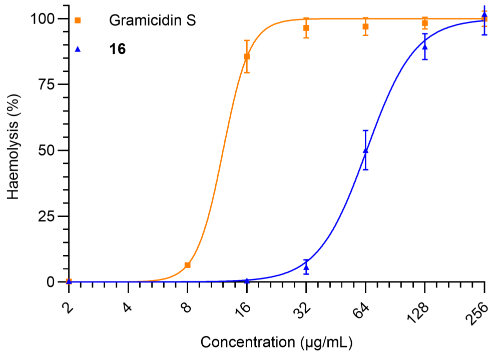
Gramicidin S
16
Haemolysis (%)
100
75
50
25
0
2
4
8
16
32
64
128
256
Concentration (μg/mL)

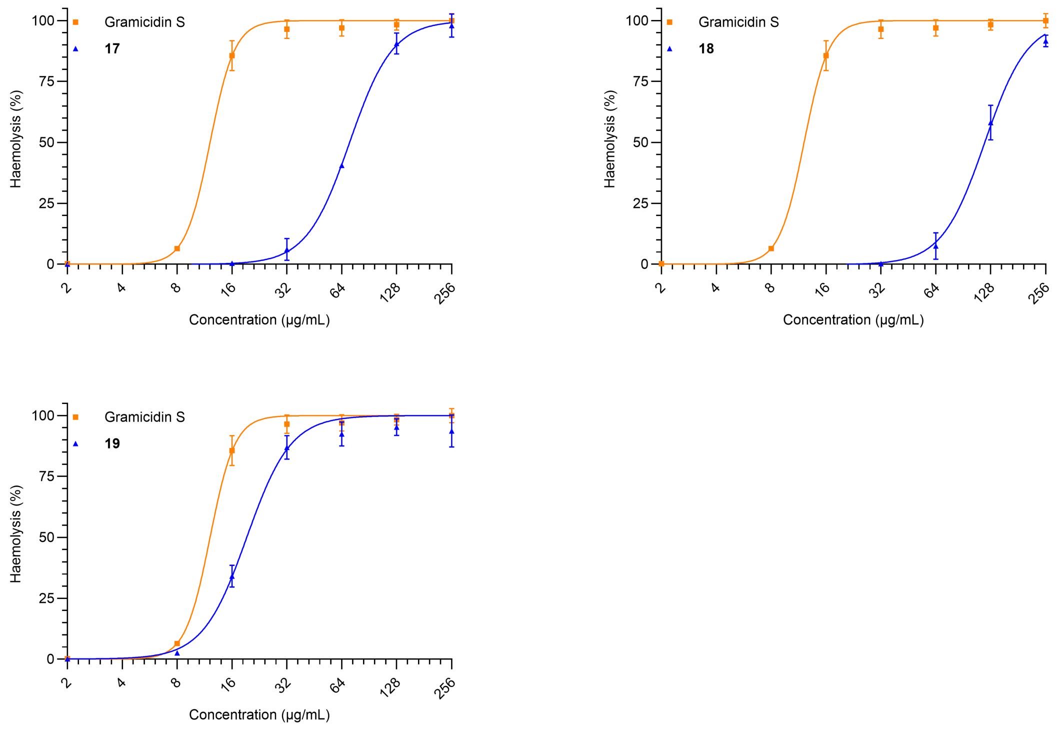

Figure S1. Normalised haemolysis (%) of human red blood cells in response to increasing concentrations of gramicidin S and peptides **1** - **19**.

3. Mass Spectrometry

Table S2. HRMS data for gramicidin S and peptides **1-19**.

Peptide	Mass (Calc'd)	Mass (found)
**Gramicidin S**	$[M + H]^+$ = 1141.7139	$[M + H]^+$ = 1141.7134
**1**	$[M + H]^+$ = 1169.7450	$[M + H]^+$ = 1169.7451
**2**	$[M + H]^+$ = 1230.7403	$[M + H]^+$ = 1230.7404
**3**	$[M + H]^+$ = 1269.7512	$[M + H]^+$ = 1269.7501
**4**	$[M + H]^+$ = 1319.7668	$[M + H]^+$ = 1319.7650
**5**	$[M + H]^+$ = 1358.7777	$[M + H]^+$ = 1358.7774
**6**	$[M + 2H]^+$ = 1398.7964	$[M + 2H]^+$ = 1398.7955
**7**	$[M + H]^+$ = 1178.7777	$[M + H]^+$ = 1178.7779
**8**	$[M + H]^+$ = 1239.7730	$[M + H]^+$ = 1239.7740
**9**	$[M + H]^+$ = 1150.7464	$[M + H]^+$ = 1150.7457
**10**	$[M + H]^+$ = 1239.7730	$[M + H]^+$ = 1239.7734
**11**	$[M + H]^+$ = 1328.7995	$[M + H]^+$ = 1328.7989
**12**	$[M + H]^+$ = 1155.7294	$[M + H]^+$ = 1155.7292
**13**	$[M + H]^+$ = 1169.7450	$[M + H]^+$ = 1169.7450
**14**	$[M + 2H]^+$ = 1142.7215	$[M + 2H]^+$ = 1142.7132
**15**	$[M + H]^+$ = 1192.7682	$[M + H]^+$ = 1192.7684
**16**	$[M + H]^+$ = 1178.7526	$[M + H]^+$ = 1178.7525
**17**	$[M + H]^+$ = 1178.7526	$[M + H]^+$ = 1178.7519
**18**	$[M + H]^+$ = 1164.7369	$[M + H]^+$ = 1164.7371
**19**	$[M + H]^+$ = 1178.7777	$[M + H]^+$ = 1178.7771

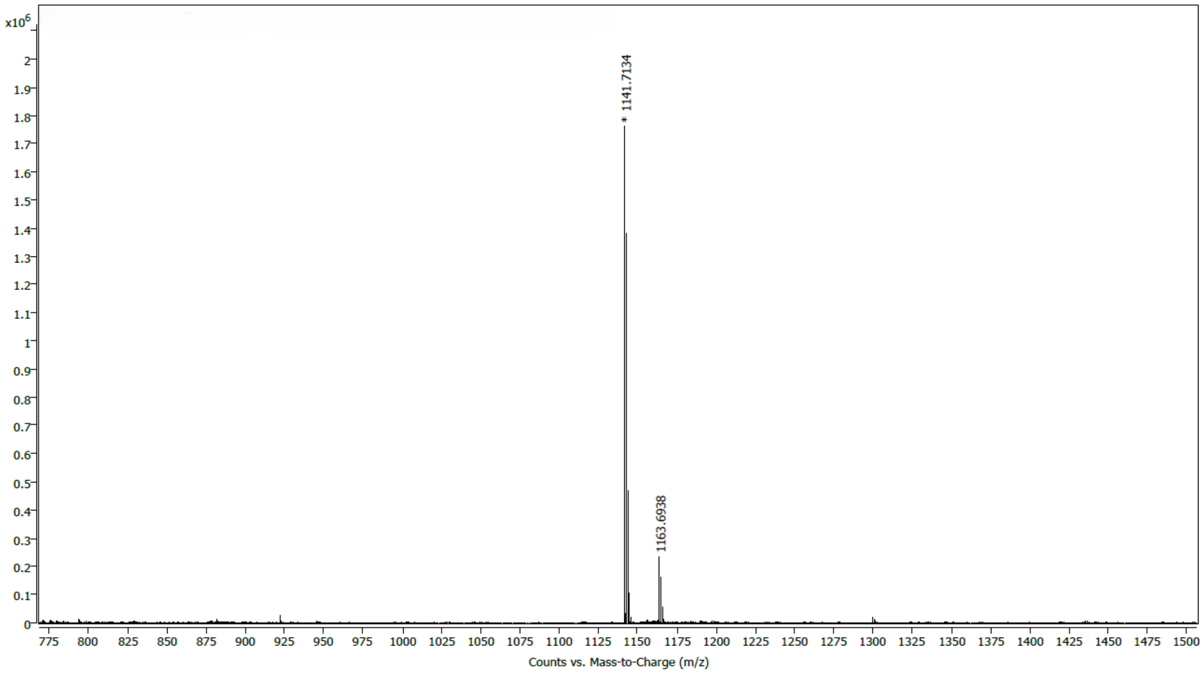

Figure S2. HRMS of Gramicidin S. $[M + H]^+$ calc'd = 1141.7139, $[M + H]^+$ found = 1141.7134.

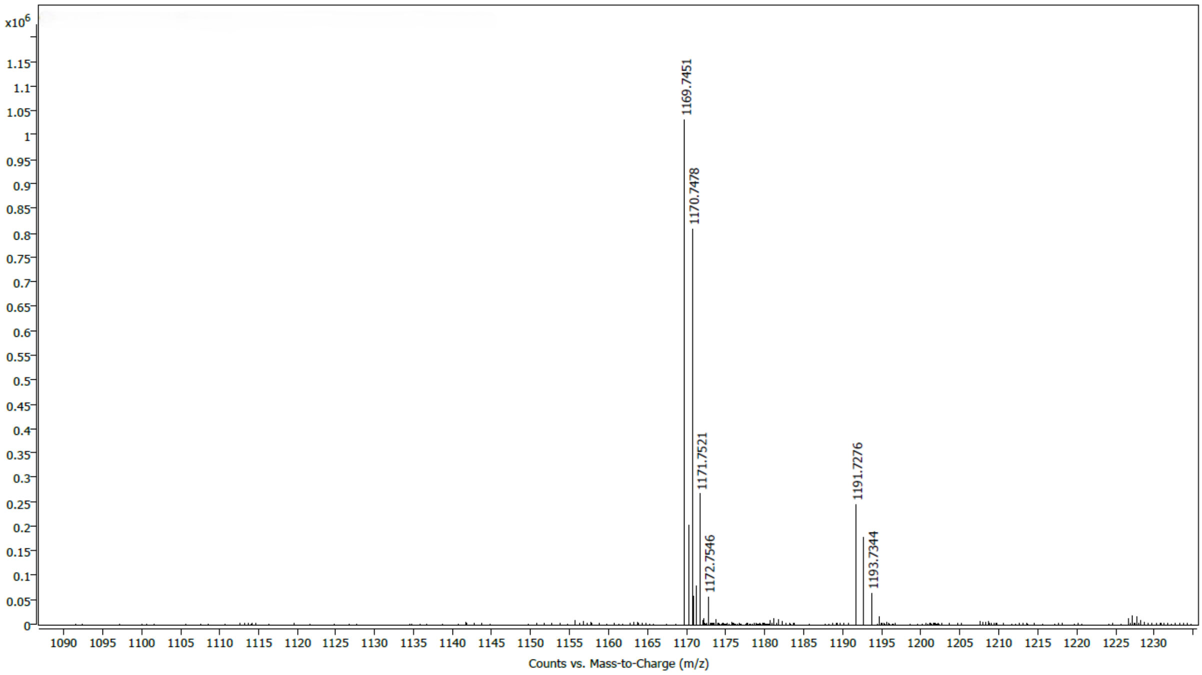

Figure S3. HRMS of peptide **1**. $[M + H]^+$ calc'd = 1169.7450, $[M + H]^+$ found = 1169.7451.

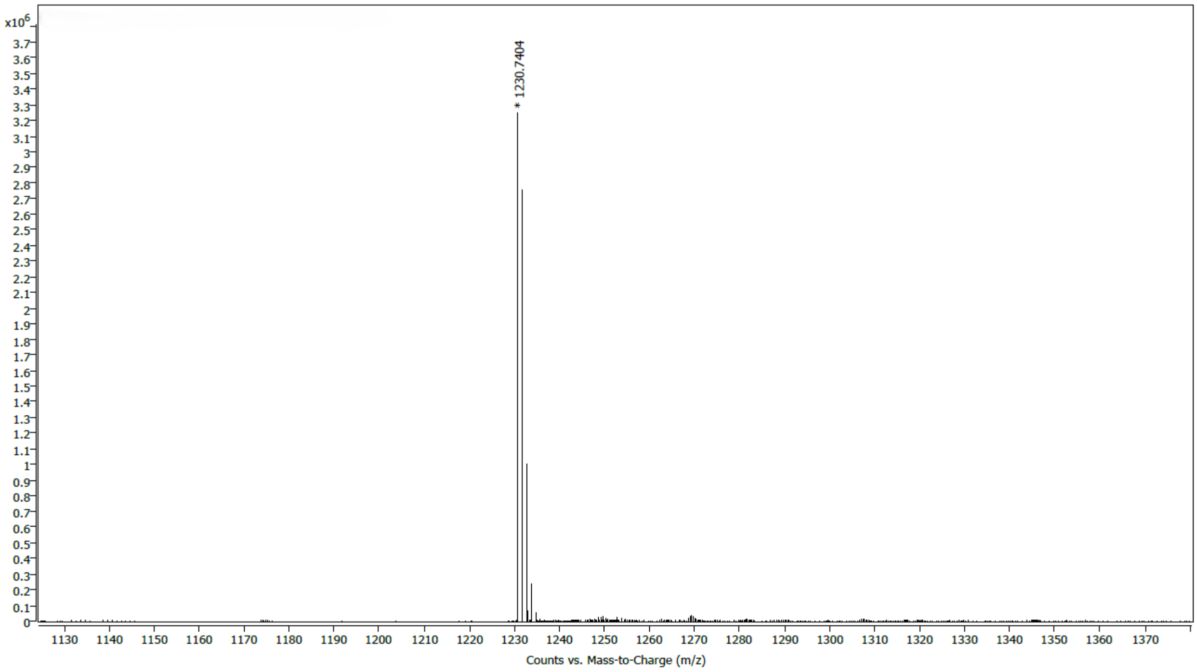

Figure S4. HRMS of peptide **2**. $[M + H]^+$ calc'd = 1230.7403, $[M + H]^+$ found = 1230.7404.

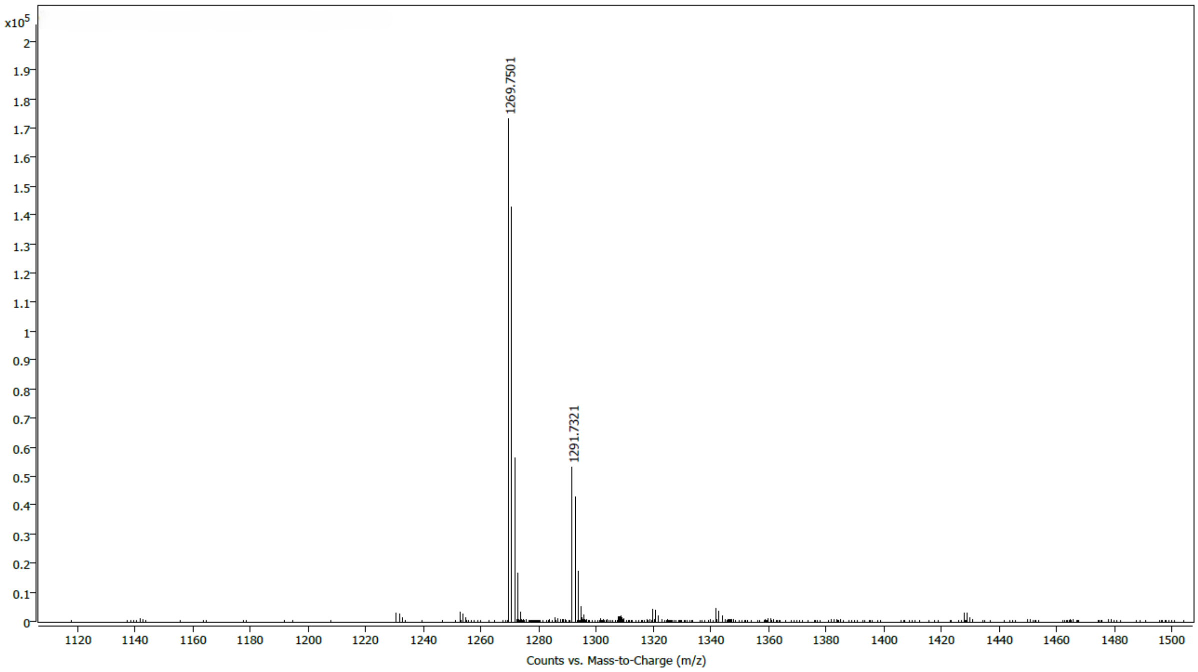

Figure S5. HRMS of peptide **3**. $[M + H]^+$ calc'd = 1269.7512, $[M + H]^+$ found = 1269.7501.

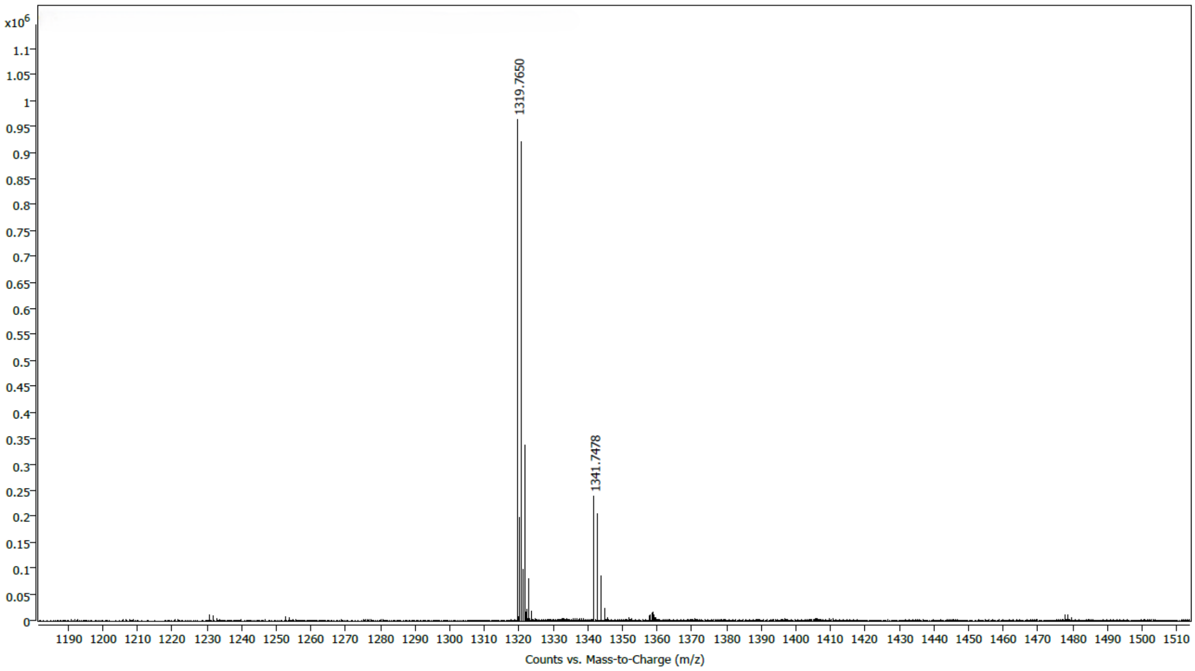

Figure S6. HRMS of peptide **4**. $[M + H]^+$ calc'd = 1319.7668, $[M + H]^+$ found = 1319.7650.

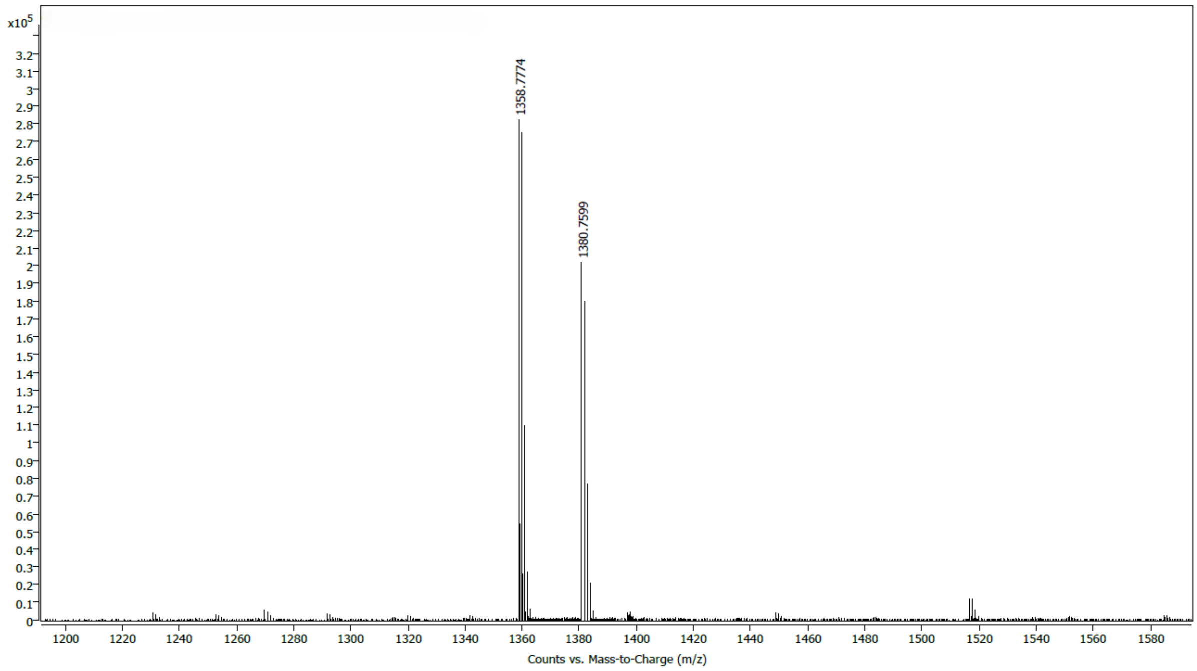

Figure S7. HRMS of peptide **5.** [M + H]$^{+}$ calc'd = 1358.7777, [M + H]$^{+}$ found = 1358.7774.

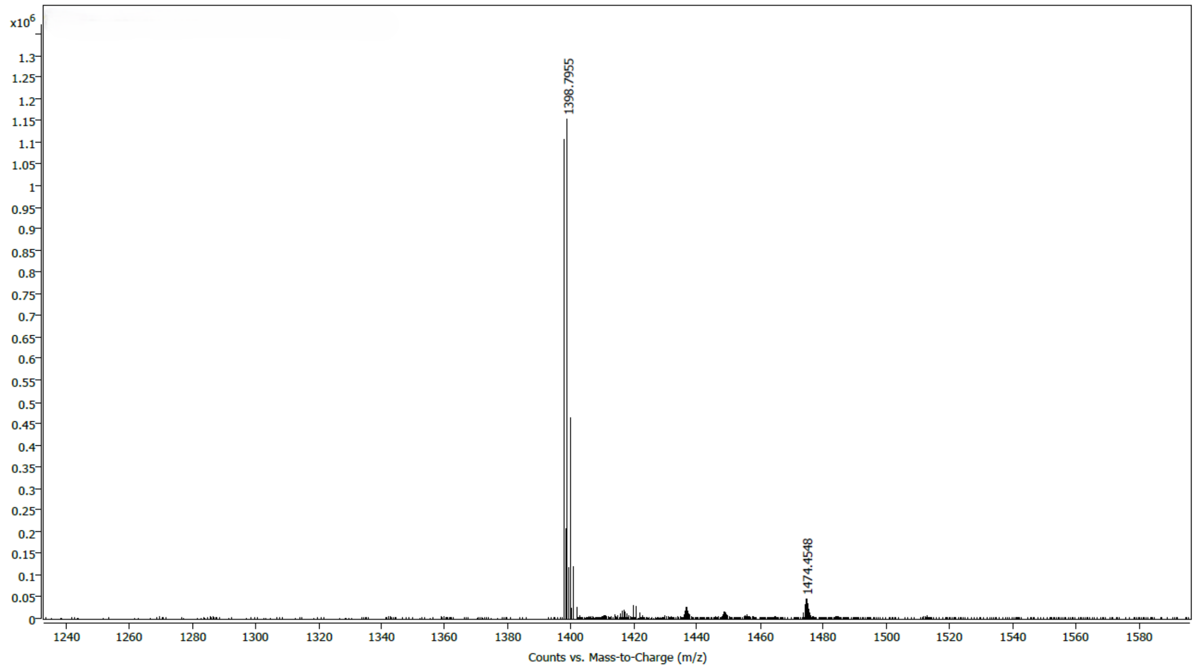

Figure S8. HRMS of peptide **6.** [M + 2H]$^{+}$ calc'd = 1398.7964, [M + 2H]$^{+}$ found = 1398.7955.

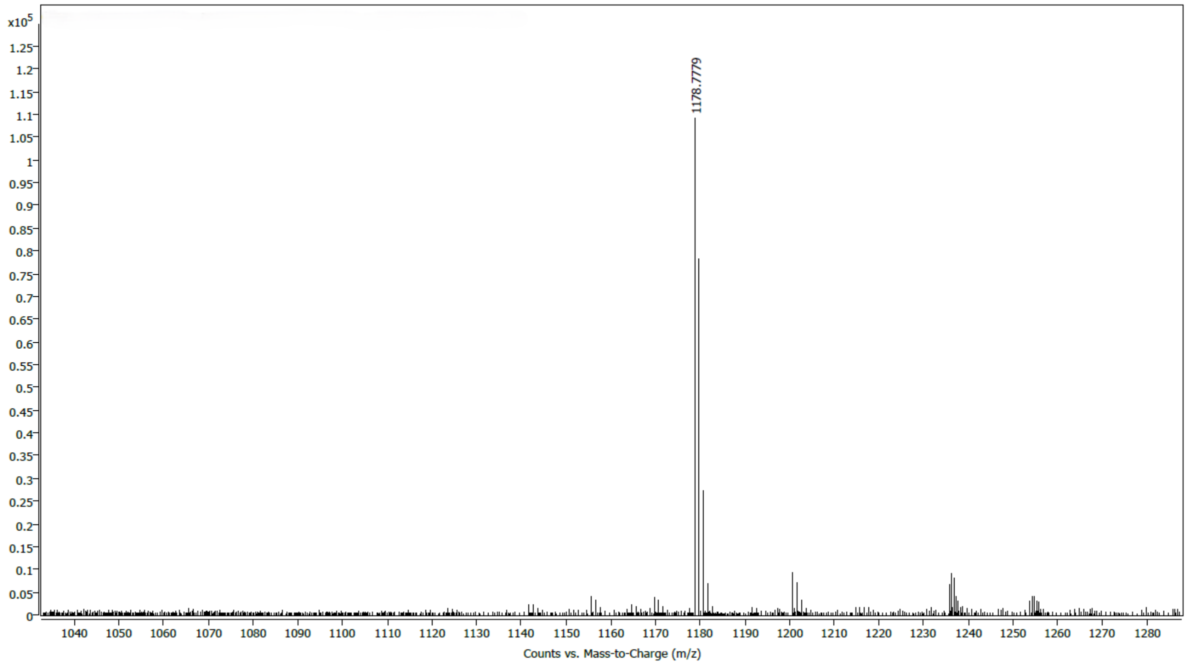

Figure S9. HRMS of peptide **7.** $[M + H]^+$ calc'd = 1178.7777, $[M + H]^+$ found = 1178.7779.

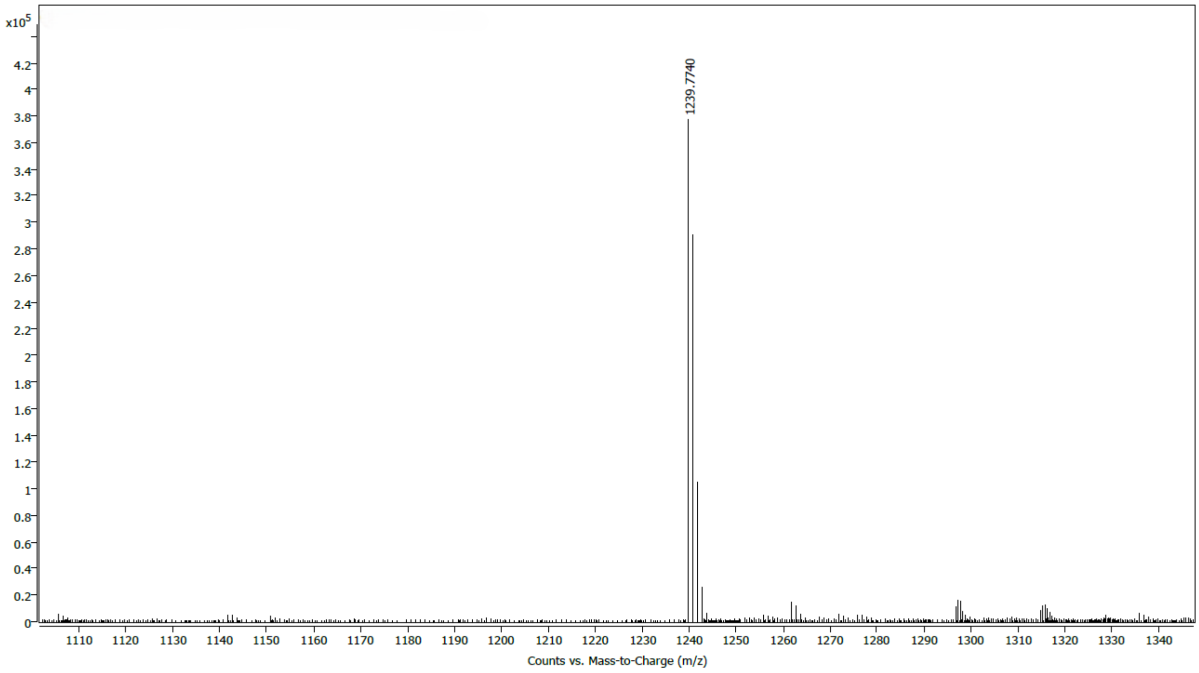

Figure S10. HRMS of peptide **8.** $[M + H]^+$ calc'd = 1239.7730, $[M + H]^+$ found = 1239.7740.

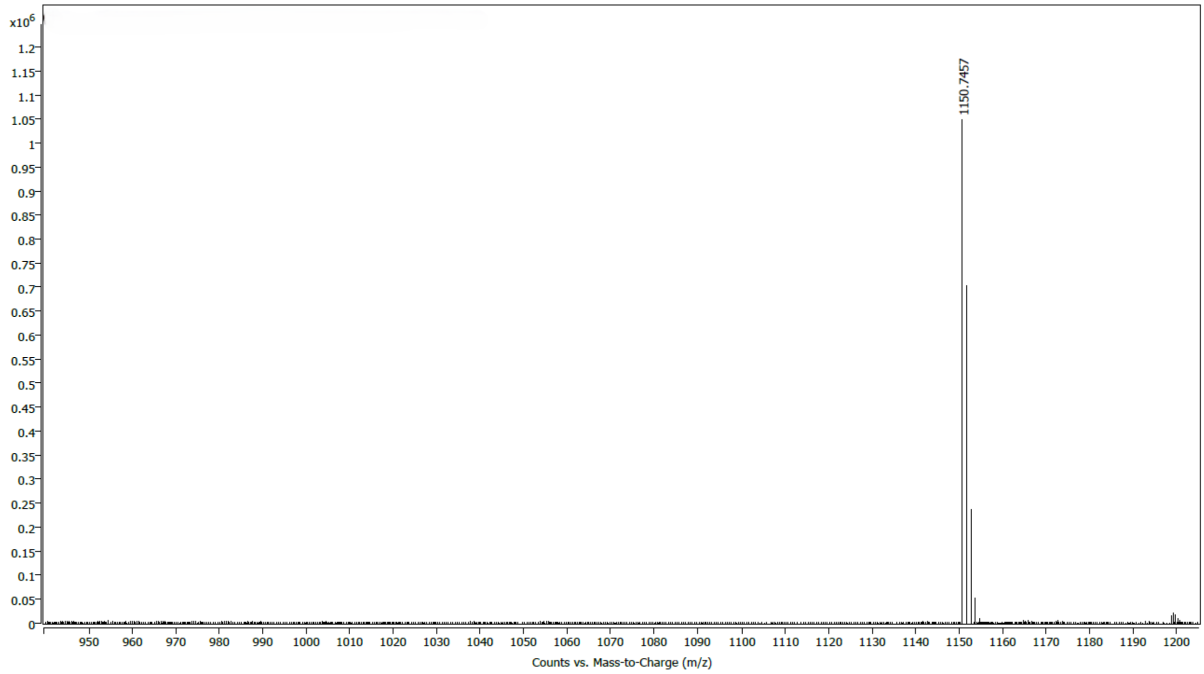

Figure S11. HRMS of peptide **9**. $[M + H]^+$ calc'd = 1150.7464, $[M + H]^+$ found = 1150.7457.

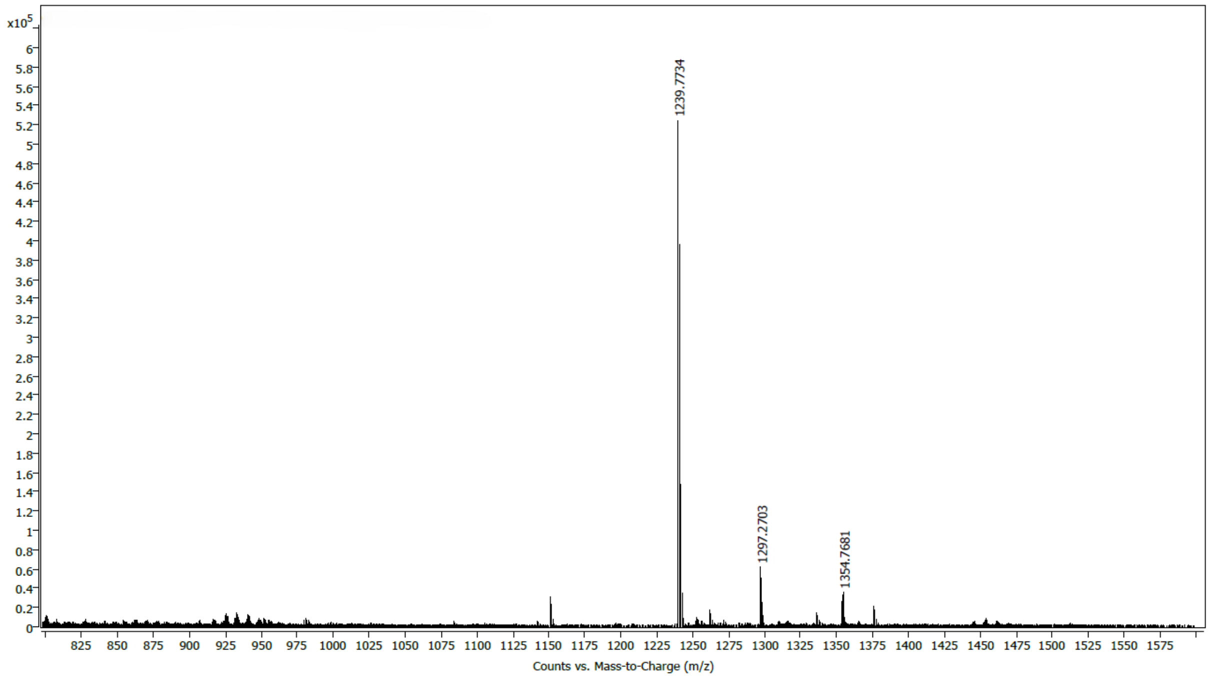

Figure S12. HRMS of peptide **10**. $[M + H]^+$ calc'd = 1239.7730, $[M + H]^+$ found = 1239.7734.

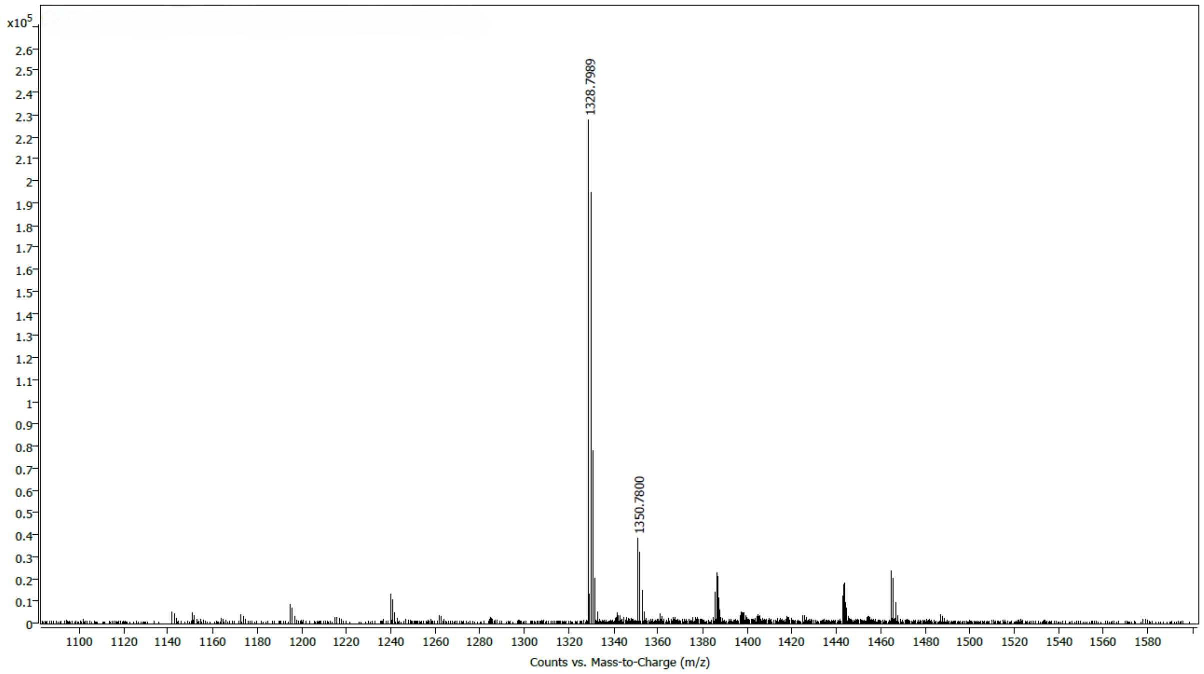

Figure S13. HRMS of peptide **11**. $[M + H]^+$ calc'd = 1328.7995, $[M + H]^+$ found = 1328.7989.

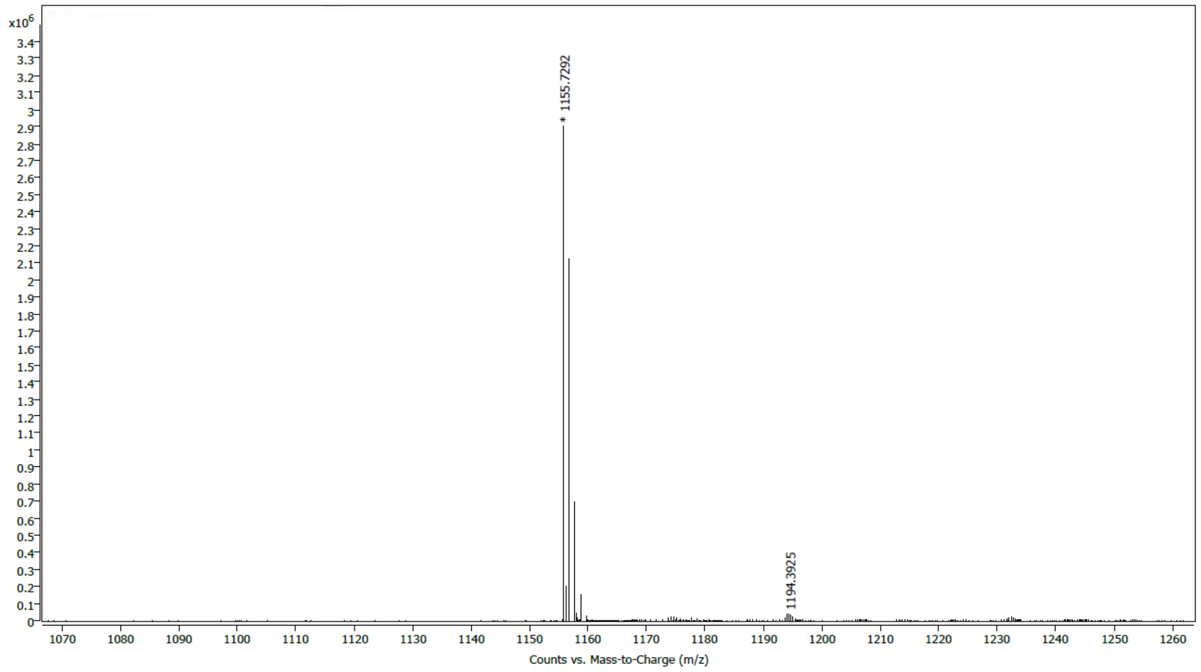

Figure S14. HRMS of peptide **12**. $[M + H]^+$ calc'd = 1155.7294, $[M + H]^+$ found = 1155.7292.

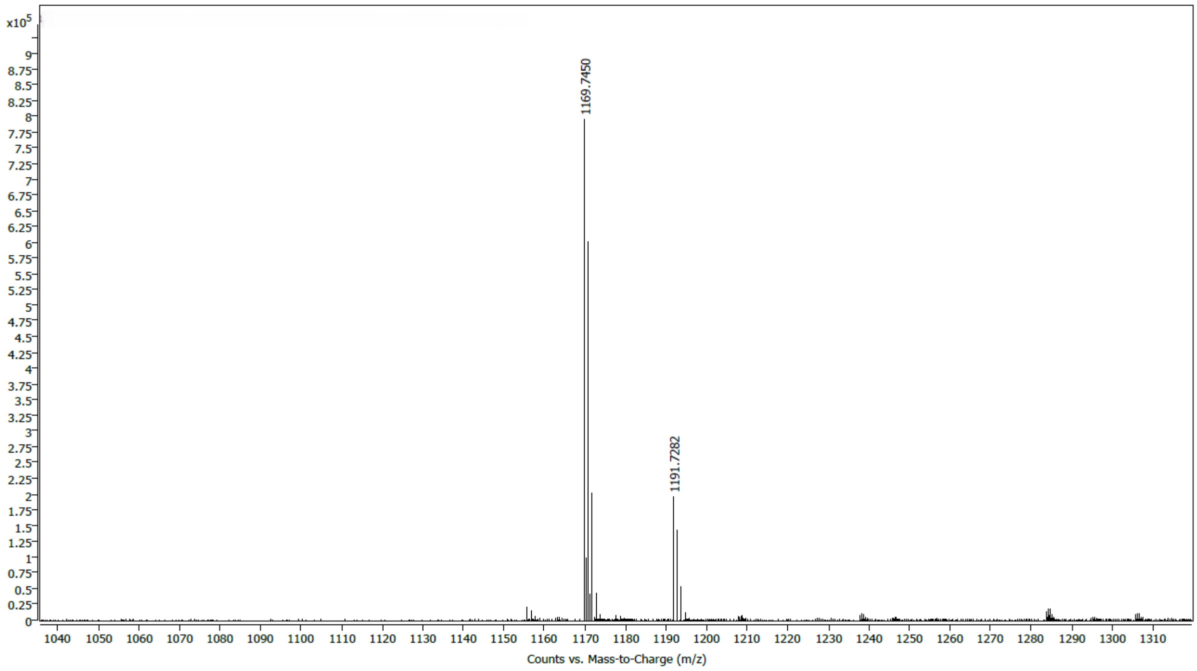

Figure S15. HRMS of peptide **13**. [M + H]+ calc'd = 1169.7450, [M + H]+ found = 1169.7450.

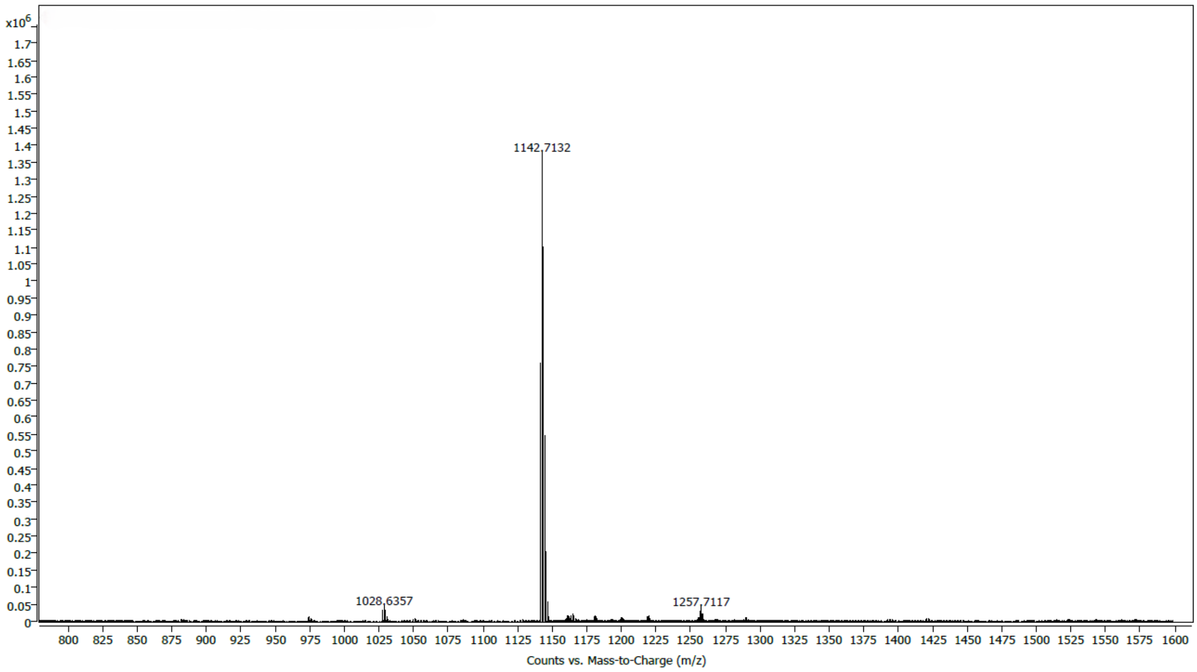

Figure S16. HRMS of peptide **14**. [M + 2H]+ calc'd = 1142.7215, [M + 2H]+ found = 1142.7132.

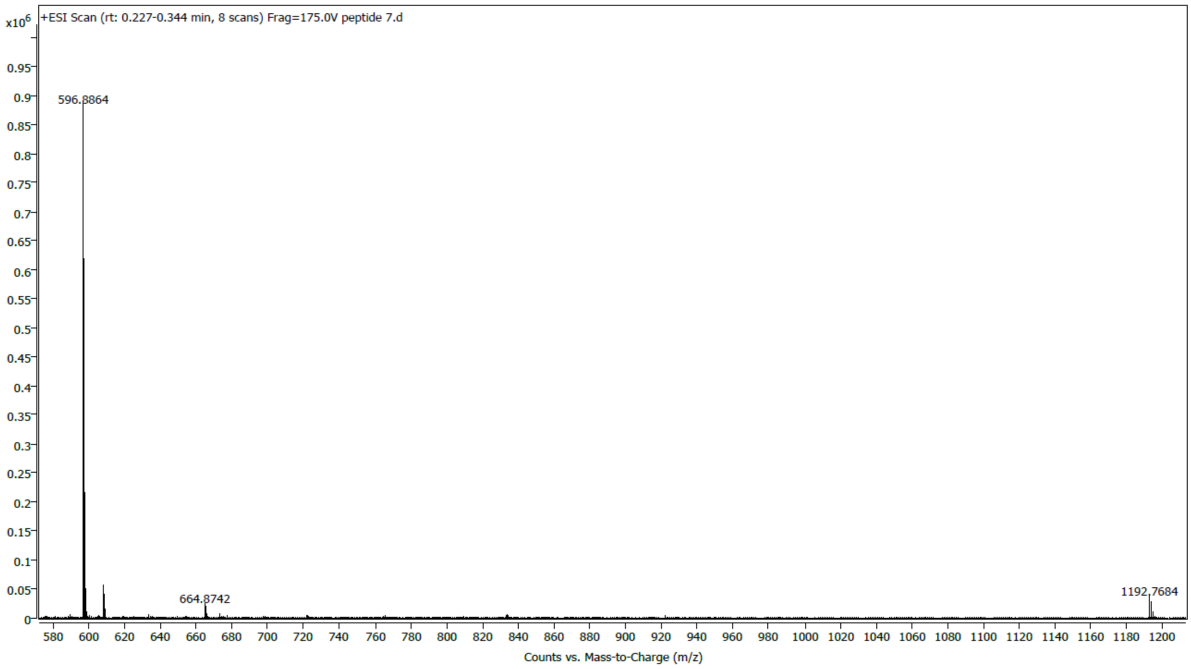

Figure S17. HRMS of peptide **15**. [M + H]+ calc'd = 1192.7682, [M + H]+ found = 1192.7684.

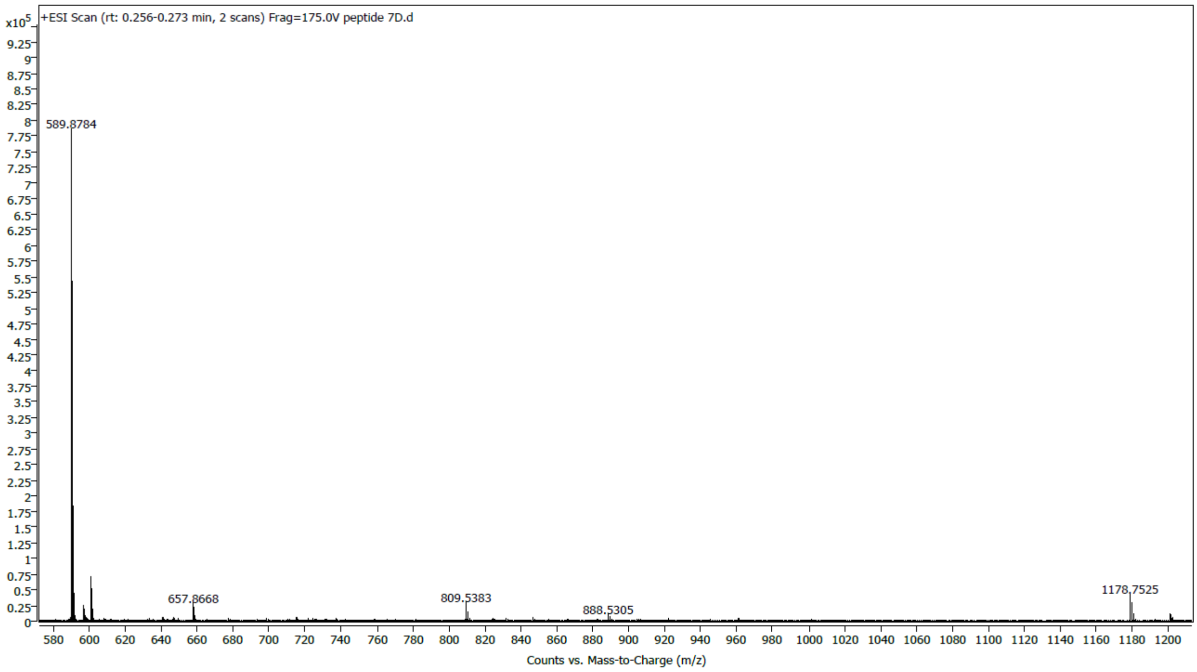

Figure S18. HRMS of peptide **16**. [M + H]+ calc'd = 1178.7526, [M + H]+ found = 1178.7525.

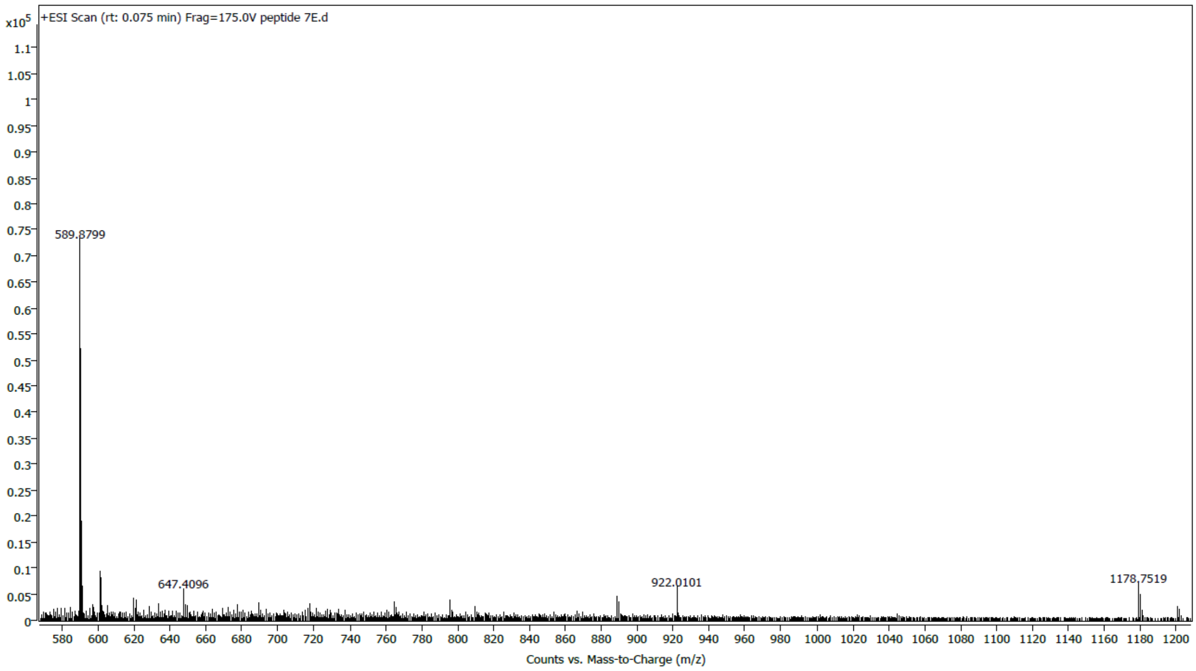

Figure S19. HRMS of peptide **17**. [M + H]+ calc'd = 1178.7526, [M + H]+ found = 1178.7519.

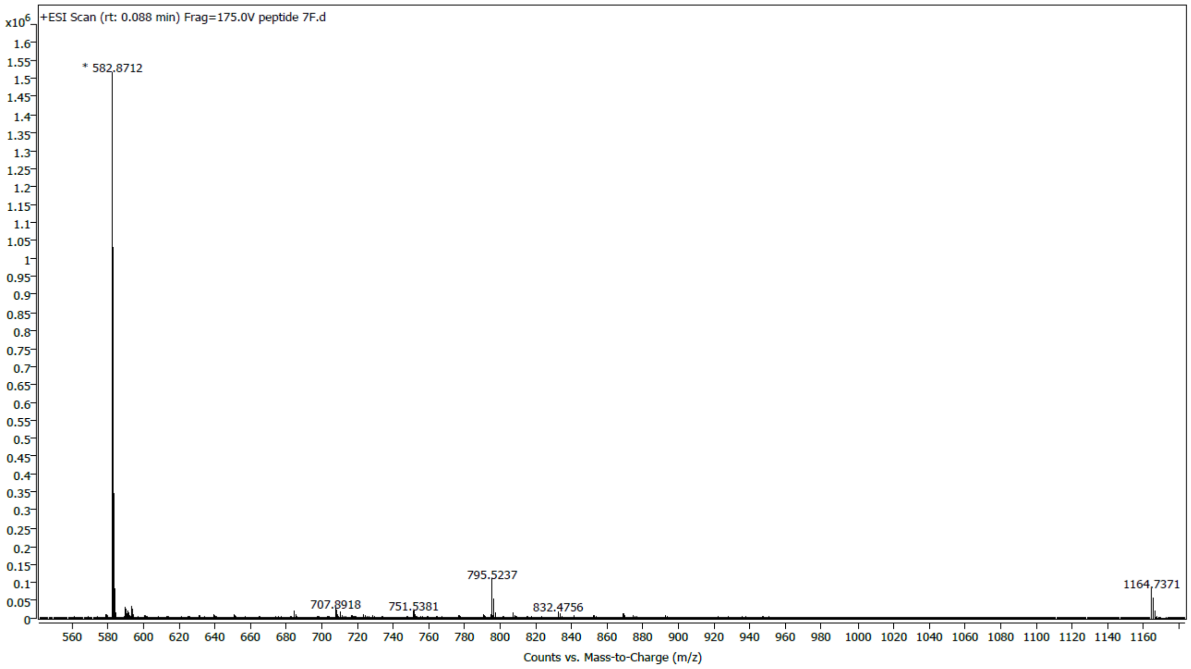

Figure S20. HRMS of peptide **18**. [M + H]+ calc'd = 1164.7369, [M + H]+ found = 1164.7371.

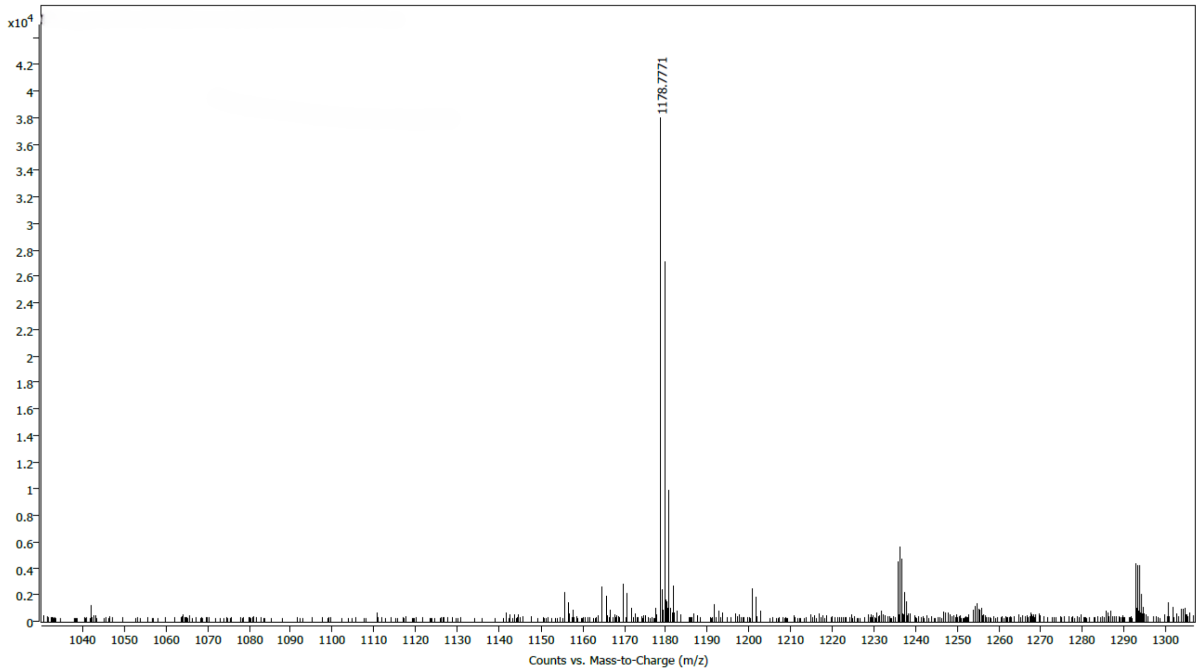

Figure S21. HRMS of peptide **19**. [M + H]+ calc'd = 1178.7777, [M + H]+ found = 1178.7771.

4. RP-HPLC

Table S3. Analytical RP-HPLC for gramicidin S and peptides **1-19** was performed on a HP Series 1100 with a Phenomenex Kinetex 2.6 µM C_{18} column (150 × 4.60 mm) at a flow rate of 0.7 mLmin^{-1} using ACN/TFA (100:0.0008 v/v) and water/TFA (100:0.001 v/v) as organic and aqueous buffers, respectively.

Peptide:	RP-HPLC retention time (min):
**Gramicidin S**	18.58
**1**	19.94
**2**	18.78
**3**	18.79
**4**	18.58
**5**	18.68
**6**	18.69
**7**	16.92
**8**	16.57
**9**	16.22
**10**	16.50
**11**	16.59
**12**	19.38
**13**	19.99
**14**	18.70
**15**	16.47
**16**	16.24
**17**	16.32
**18**	16.03
**19**	17.15

Note: All peptides >95% pure by HPLC analysis.

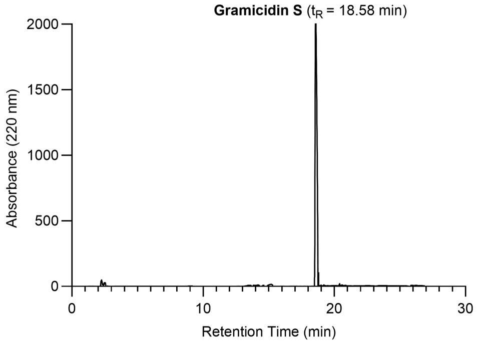
Gramicidin S (t_R = 18.58 min)
Absorbance (220 nm)
Retention Time (min)
0
500
1000
1500
2000
0
10
20
30

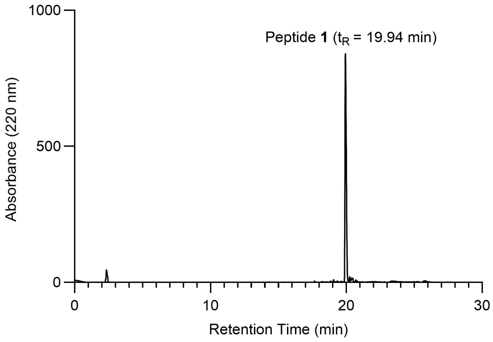
Peptide **1** (t_R = 19.94 min)
Absorbance (220 nm)
Retention Time (min)
0
500
1000
0
10
20
30

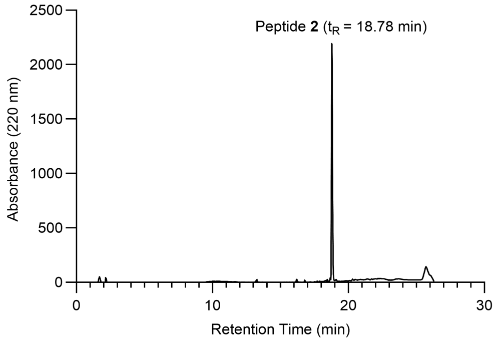
Peptide **2** (t_R = 18.78 min)
Absorbance (220 nm)
Retention Time (min)
0
500
1000
1500
2000
2500
0
10
20
30

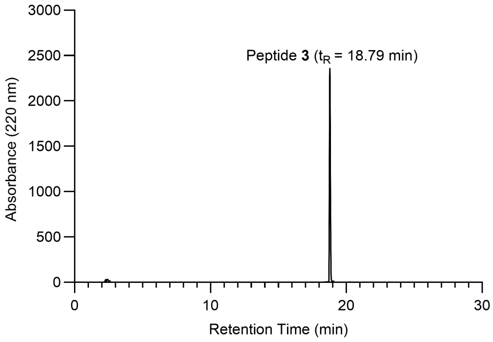
Peptide **3** (t_R = 18.79 min)
Absorbance (220 nm)
Retention Time (min)
0
500
1000
1500
2000
2500
3000
0
10
20
30

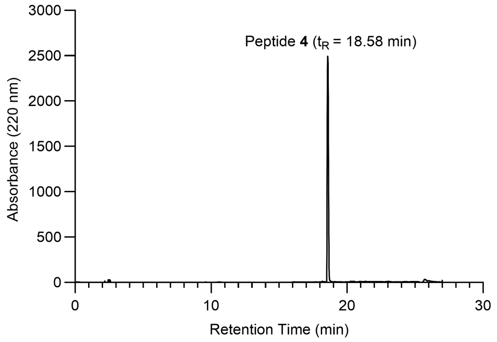
Peptide **4** (t_R = 18.58 min)
Absorbance (220 nm)
Retention Time (min)
0
500
1000
1500
2000
2500
3000
0
10
20
30

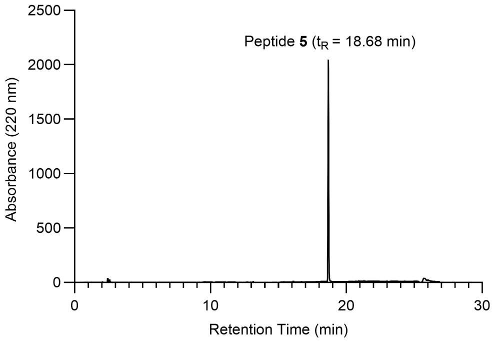
Peptide **5** (t_R = 18.68 min)
Absorbance (220 nm)
Retention Time (min)
0
500
1000
1500
2000
2500
0
10
20
30

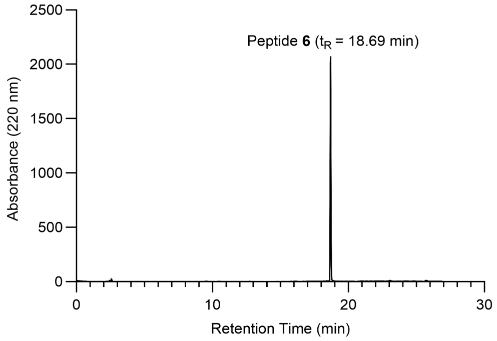
Peptide **6** (t_R = 18.69 min)
Absorbance (220 nm)
Retention Time (min)
0
500
1000
1500
2000
2500
0
10
20
30

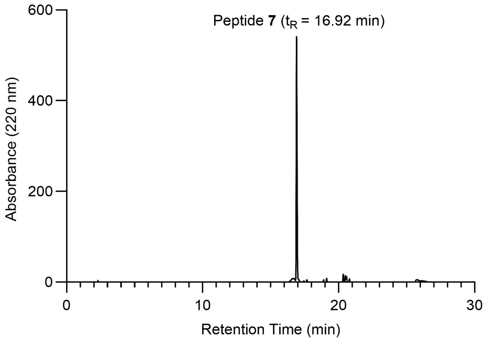
Peptide **7** (t_R = 16.92 min)
Absorbance (220 nm)
Retention Time (min)
0
200
400
600
0
10
20
30

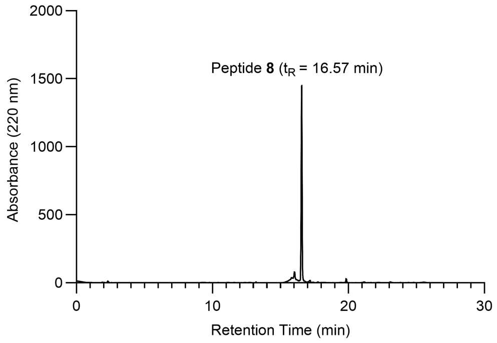
Peptide **8** (t_R = 16.57 min)
Absorbance (220 nm)
Retention Time (min)

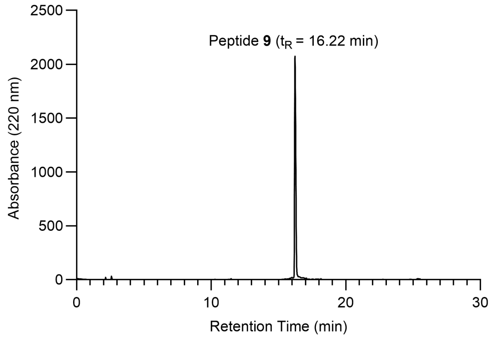
Peptide **9** (t_R = 16.22 min)
Absorbance (220 nm)
Retention Time (min)

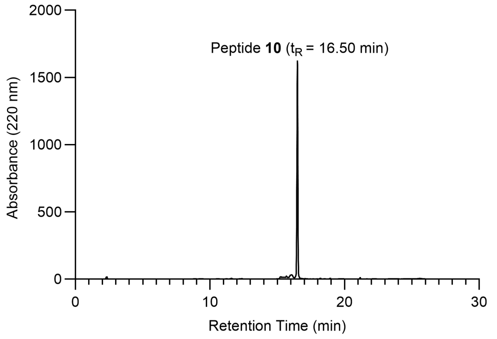
Peptide **10** (t_R = 16.50 min)
Absorbance (220 nm)
Retention Time (min)

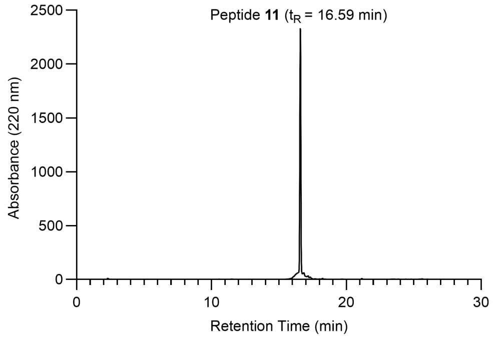
Peptide **11** (t_R = 16.59 min)
Absorbance (220 nm)
Retention Time (min)

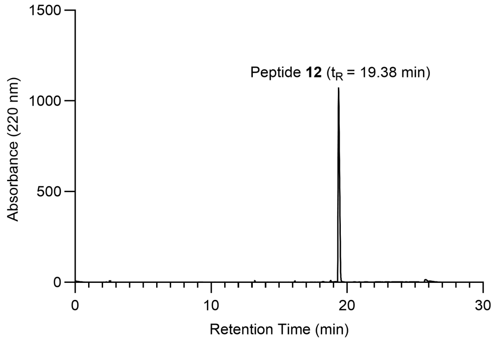
Peptide **12** (t_R = 19.38 min)
Absorbance (220 nm)
Retention Time (min)

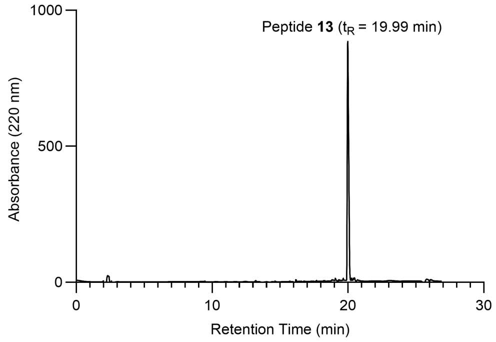
Peptide **13** (t_R = 19.99 min)
Absorbance (220 nm)
Retention Time (min)

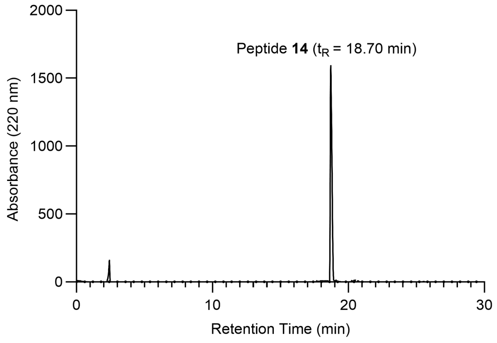
Peptide **14** (t_R = 18.70 min)
Absorbance (220 nm)
Retention Time (min)

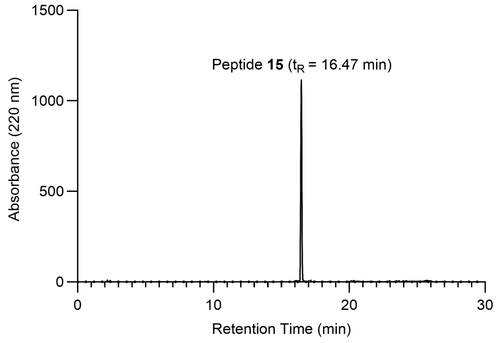
Peptide **15** (t_R = 16.47 min)
Absorbance (220 nm)
Retention Time (min)

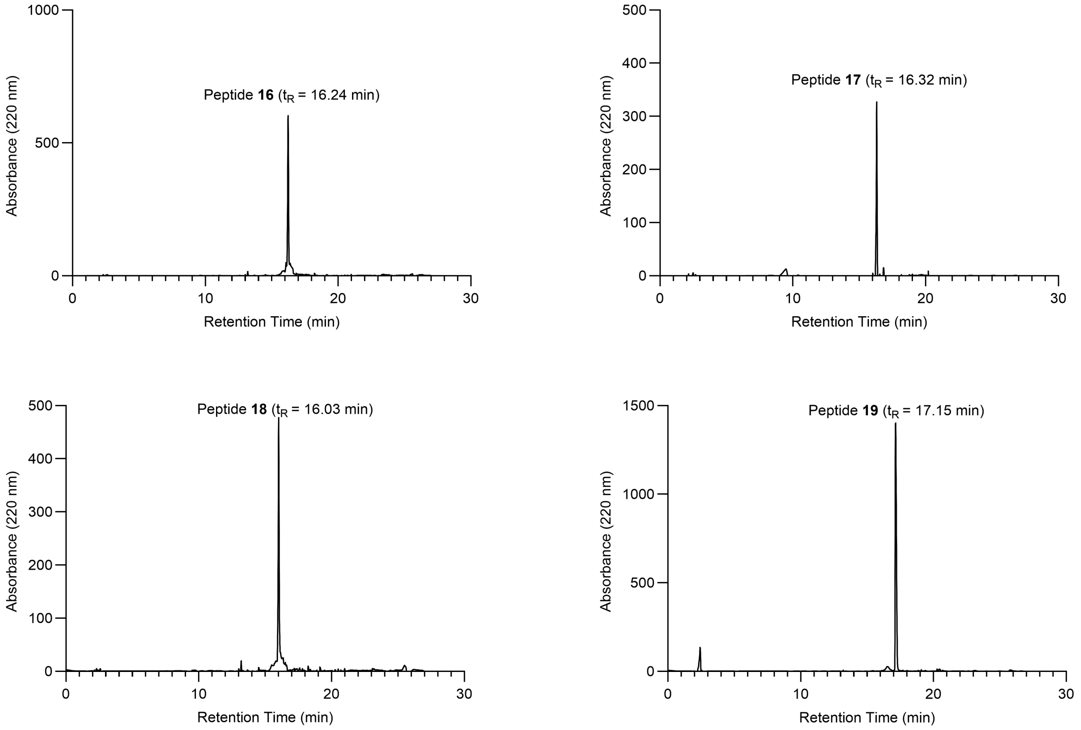

Figure S22. Analytical RP-HPLC traces for gramicidin S and peptides **1-19**.
